# Integrating SARIMA Forecasting and Metabolomics to Decode Seasonal Chemotype Variation in *Ayapana triplinervis*

**DOI:** 10.1021/acsomega.5c10960

**Published:** 2025-11-27

**Authors:** Jonathan Lopes de Matos, Lucas de Sena Pantoja, Kryssia Jarina Tavares Monteiro, Lethicia Barreto Brandão, Victor Hugo de Souza Marinho, Irlon Maciel Ferreira, Fábio Rodrigues de Oliveira, Ryan da Silva Ramos, Alex Bruno Lobato Rodrigues

**Affiliations:** † Department of Biological and Health Sciences, 74364 Federal University of Amapá, Maapá, AP 68902-280, Brazil; ‡ Department of Exact and Technological Sciences, Federal University of Amapá, Macapá, AP 68902-280, Brazil

## Abstract

Although *Ayapana triplinervis* has been extensively investigated for its phytochemical composition and pharmacological potential, the effects of climatic variability on its essential oil metabolism remain poorly understood. This study bridges this knowledge gap by integrating climate forecasting with metabolomic profiling to elucidate seasonal and morphotype-specific chemical variations in the essential oils of *A. triplinervis* from the Brazilian Amazon. Precipitation patterns predicted by a SARIMA model delineated distinct hydrological phases, guiding four strategic sampling periods throughout 2024. Combined ^1^H NMR, GC–MS, and multivariate analyses revealed pronounced seasonal metabolic shifts. Morphotype B maintained a chemically stable profile dominated by Thymohydroquinone Dimethyl Ether (THDE), whereas Morphotype A exhibited greater metabolic flexibility, shifting toward *cis*-caryophyllene during the transitional period between the Amazonian summer and winter. Variations in rainfall intensity modulated the balance between oxygenated phenylpropanoids and sesquiterpenes, indicating divergent adaptive strategies between morphotypes. This integrated forecast–metabolomics framework demonstrates how climatic predictability can be harnessed to anticipate metabolic adjustments in tropical aromatic species, offering new perspectives for chemotaxonomic differentiation, quality assurance, and sustainable utilization of Amazonian biodiversity.

## Graphical abstract

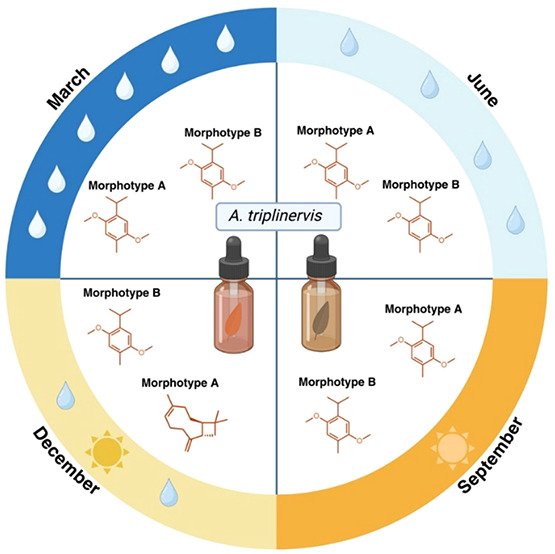

## Introduction

Among the species of medicinal interest in the Asteraceae family, *Ayapana triplinervis* (Vahl) RM King & H. Rob. can be found in Brazil, Ecuador, Peru, Puerto Rico, and Guianas, and has also been adapted in other countries such as India and Vietnam.^1^ Morphoanatomical studies have shown that the variant popularly known as “Japana-branca” has a green stem, while the variant “Japana-roxa” presents a purple stem, in addition to differences in leaf vein, leaf base, internode length, branching, and a greater number of leaf shoots in “Japana-branca”.^2^

*A. triplinervis* is used by traditional communities in South America and Asia in various preparations (tea, decoction, tinctures or baths) for treating viral diseases, respiratory and gynecological ailments, and in spiritual practices. In West Bengal (India), the leaves are used to combat dysentery and bloody enteritis,^3^ while records from Madagascar and the Mascarene Islands (Africa) report uses to relieve stomach burning, indigestion, diarrhea, insomnia, nausea, ulcer, vomiting and flu, as well as astringent, emollient and febrifuge actions.^4^ In the Amazon context, researchers have reported the leaves are employed for mystical-religious purposes, as well as to treat constipation, headache, cough, and respiratory diseases.^5^ In the Amapá (Brazil) region, there are reports of its use against cholera, tetanus, and leptospirosis,^6^ and on islands such as Mauritius, both for gastrointestinal applications (vomiting, diarrhea, stomach aches, and colitis) and for relieving abdominal distension.^7^ In India, leaf extracts have been used to control menstrual bleeding.^8^

Regarding phytochemical studies and ethnopharmacological validation, although some authors have differentiated the chemical composition between morphotypes based on biological activity, some studies have evaluated the correlation between traditional medicinal uses and the presence of secondary metabolites. Petroleum ether extracts have demonstrated antinociceptive and anti-inflammatory potential, suggesting the presence of lipophilic bioactive compounds.^9^ Isolated compounds, such as 7-methoxycoumarin, exhibited antimelanogenic activity and inhibition of B16 melanoma cell lines, while 6,7-methylenedioxy coumarin showed low oral toxicity and antinociceptive effects.^10^ Methanolic extracts have also demonstrated antiulcerogenic effects in animal models, possibly through protecting the gastric mucosa.^11^ Moreover, hydroalcoholic extracts are associated with anxiolytic, antidepressant, antinociceptive, and antioxidant effects in animal models, indicating their action on neurochemical systems and oxidative stress parameters.^12^ In studies on antineoplastic activity, aqueous and ethanolic fractions demonstrated antimitotic, apoptotic, and antineoplastic potential against Ehrlich carcinoma, both *in vivo* and *in vitro*,^13^ whereas methanolic extracts showed hypocholesterolemic, antioxidant, antiproliferative, and anticancer effects in cell lines.^14^

*A. triplinervis* has garnered considerable attention for its diverse therapeutic and industrial applications. One promising approach involves the green synthesis of silver nanoparticles using extracts of *A. triplinervis*. The biosynthesized silver nanoparticles demonstrated potent antimicrobial activity against common wound pathogens, including *Escherichia coli*, *Staphylococcus aureus*, and *Pseudomonas aeruginosa*. Compared to the crude plant extract, the nanoparticles also exhibited enhanced antioxidant and anti-inflammatory properties while maintaining low cytotoxicity, which suggests good biocompatibility for potential wound-healing applications.^15^

The chemical composition and biological activity of *A. triplinervis* essential oil (AtEO) have been investigated. *In vivo* experiments revealed that the essential oil exhibits significant antinociceptive properties. In addition, AtEO demonstrated notable anti-inflammatory activity. Importantly, these antinociceptive and anti-inflammatory effects did not affect locomotor activity, indicating that sedation or muscle relaxation did not confound these findings.^16^

Hydromethanolic and petroleum extracts of *A. triplinervis* provide gastroprotective benefits in rat models, whereas methanolic extracts protect against carbon tetrachloride induced liver damage in rats. *A. triplinervis* extracts also possess antioxidant capabilities, as evidenced by free-radical scavenging assays, and preliminary in vitro studies indicate potential anticancer activity against lung cancer cell lines. There is growing evidence of insecticidal properties of essential oil nanoemulsions, which have proven effective against *Aedes aegypti* mosquitoes, and in hemostatic activities, where fresh juice and methanolic extracts promote blood coagulation in rats. Recent research has explored the development of novel pharmaceutical products, such as photoprotective formulations and antibacterial silver nanoparticles, derived from *A. triplinervis*; however, more *in vivo* and clinical studies are required to isolate specific bioactive compounds and validate their traditional uses.^17,18^

In zebrafish models, the maximum nontoxic concentration of *A. triplinervis* extract was determined to be 1.25 g/L, with no significant alterations observed in gene expression related to hepatotoxicity, cardiotoxicity, or stress biomarkers at this concentration. The lethal concentration for 50% mortality (LC_50_) was established as 3.478 g·L^–1^ for eleutheroembryos (0–96 h postfertilization) and 2.65 g·L^–1^ for larvae (3–5 days postfertilization), indicating a relatively wide safety margin^19^

In addition to medicinal applications, *A. triplinervis* extracts show promise as natural corrosion inhibitors. The aqueous leaf extract demonstrated a high inhibition efficiency of 96% at 303 K when applied to mild steel immersed in hydrochloric acid. This effect is attributed to the adsorption of phytochemicals, primarily Thymohydroquinone Dimethyl Ether (THDE) and coumarins, onto the metal surface, where both physisorption and chemisorption occur. Evidence suggests coordinate bonding between the d-orbitals of steel and the oxygen atoms in THDE, providing a robust protective layer that prevents metal oxidation.^20^

Recent antiviral research has focused on the efficacy of THDE against Zika virus (ZIKV). *In vitro* assays revealed that THDE inhibited ZIKV by blocking the internalization step of viral entry into host cells without affecting the initial binding phase. Importantly, in vivo studies using zebrafish models have reported no acute toxicity at therapeutically relevant concentrations, suggesting THDE’s potential as a safe antiviral agent. The inclusion of *A. triplinervis* in the most edition of the French Pharmacopeia underscores its recognized medicinal value and reinforces its potential as a source of novel antiviral compounds, particularly against mosquito-borne viruses.^21^

Despite a few studies on the phytochemical composition and biological activities of *A. triplinervis*, there remains a critical gap in understanding how climatic variability dynamically regulates its essential oil metabolism, particularly in the Amazon region where rainfall strongly dictates phenological and biochemical cycles.^22^ Previous works have mainly described static chemical profiles or morphotype differentiation without integrating predictive climate models or longitudinal metabolomic analyses.^23^ To date, no study has systematically linked seasonal precipitation forecasting with metabolomic responses in Amazonian chemotypes.^24^ Addressing this gap, the present study combines SARIMA-based rainfall prediction with NMR- and GC–MS-guided metabolomics to elucidate how morphotypes A and B modulate their secondary metabolism across annual hydric gradients, providing an unprecedented framework for connecting climate-driven chemotype plasticity to ecological adaptation and resource management.

This study aimed to characterize the seasonal dynamics of the metabolic profiles of the essential oils of morphotypes A (Japana-branca) and B (Japana-roxa) of *A. triplinervis* through ^1^H NMR spectroscopy, GC–MS and multivariate analysis, and to correlate these chemical variations with precipitation predictions generated by a SARIMA (Seasonal Autoregressive Integrated Moving Average) model for the same period, to identify seasonal and morphotype-specific biomarkers that guide the optimal collection time and support future pharmaceutical and industrial applications.

## Methodology

### SARIMA Forecast for Sample Collection Periods

Monthly precipitation data were obtained from the National Institute of Meteorology (INMET) portal for station A249 (Macapá, AP, Brazil), covering the period from January 31, 2004, to December 31, 2023. First, a monthly time series with a 12-month periodicity was constructed and visually inspected to identify its components in RStudio (version 2025.05.0, Posit Software, PBC).^25^ Subsequently, a Seasonal AutoRegressive Integrated Moving Average (SARIMA) model was applied, with the optimal specification automatically selected by the auto.arima­() function, which indicates the SARIMA­(2,0,2)­(1,0,1)[12] model with a nonzero mean.

Once validated, the model generated monthly precipitation forecasts for 2024, 95% confidence intervals, enabling the identification of the wettest months (March and April) and the driest months (September and October), as well as transitional periods between seasons. Based on these forecasts, sampling dates were set for March 15 (peak rainy season), June 15 (transition from rainy to dry season), September 15 (peak dry season), and December 15 (transition from dry to rainy season).

### Collection, Botanical Identification and Extraction of Essential Oils

Leaf samples of both morphotypes (A: “Japana-branca”; B: “Japana-roxa”) were collected in the Fazendinha neighborhood of Macapá (AP. Brazil), at coordinates 0°4′23″ N and 51°7′32″ W, on the 15th day of March, June, September, and December 2024 (Table 1). Each sample was submitted to the Herbário Amapaense at the Instituto de Pesquisas Científicas e Tecnológicas do Amapá (IEPA) and deposited under voucher codes ABLR001-HAMAB and ABLR002-HAMAB. Fresh leaves from each morphotype were separately subjected to hydrodistillation using a Clevenger apparatus at 100 °C for 2 h. The extracted essential oils were collected in amber vials and stored at −4 °C, protected from light, until further analyses.^26^

**Table 1:** Monthly Total Precipitation in Macapá, Amapá, Brazil^25^

Sampling period (2024)	Monthly total precipitation (mm)	Seasonal characteristic
March	366	Amazonian winter
June	273.4	Transition from winter to summer
September	0.0	Amazonian summer
December	39.8	Transition from summer to winter

### ^1^H Nuclear Magnetic Resonance Analysis

^1^H NMR analyses were performed on a Bruker Avance III 500 MHz spectrometer equipped with a 5 mm probe. For sample preparation, 5 mg of each essential oil was dissolved in 600 μL of deuterated chloroform containing trimethylsilane (TMS) as an internal calibration standard. Initial processing of free induction decay (FID) signals was performed using NMRProcFlow (version 1.4.26), employing the Metabolic Fingerprint strategy to standardize and compare spectra obtained during the four sampling months.^27^

### Qualitative Analysis of Chemical Groups

An exploratory analysis of chemical groups observed in the hydrogen NMR spectra (^1^H NMR) was conducted through the stratification and the chemical shifts, intensities, and relative areas of the signals (Table 2). Principal Component Analysis (PCA) was then applied, and the corresponding scores and loadings were exported for customized visualization of seasonal patterns and morphotype differences. The number of principal components retained was determined according to Kaiser’s criterion.^28^

**Table 2:** Qualitative Characterization of Chemical Groups by ^1^H NMR^29^

Groups	Chemical Shift (ppm)	Assignments
Aliphatic	0.5–1.5	–CH_n_; −CH_n_
Allylic/Methylenic	1.5–3.0	CH_n_–C=O; CH_n_–N; Ar–CH_n_; Ar–CH_n_-
Oxygenated	3.0–4.5	CH_n_–C=O; −CH_n_–O–; −CH_n_–N–
Vinyl	4.5–6.0	Ph–O–CH_n_; HC=C– (non conjugated)
Aromatic	6.0–9.0	Ph-H; Ph–CH=O
Aldehydic	9.0–10	HCOR

### Gas Chromatography–Mass Spectrometry Analysis

The chemical composition of the essential oils was determined by gas chromatography–mass spectrometry (GC–MS) using a Shimadzu GCMS-QP 5050A instrument coupled to a DB-5HT capillary column (J & W Scientific), 30 m in length, 0.32 mm internal diameter, and 0.10 μm film thickness, with nitrogen as the carrier gas. GC-MS operating conditions included a column head pressure of 56.7 kPa, a split ratio of 1:20, a carrier gas flow rate of 1.0 mL·min^–1^ 110 °C, an injector temperature of 220 °C, and an interface temperature of 240 °C. The column oven temperature program started at 60 °C, increased at 3 °C·min^–1^ to 240 °C, and was held at that temperature for 30 min. The mass spectrometer was scanned from 29 to 400 Da at 0.5 s intervals, with an ionization energy of 70 eV. One microliter of each sample (1.0 μL) was injected at a concentration of 10,000 ppm in hexane. To calculate the Linear Retention Index (LRI), a standard *n*-alkane mixture (C8–C40, Sigma-Aldrich) was injected identical GC conditions, and the retention times for each hydrocarbon were recorded.^30^

The calculated LRI values were compared to literature references for columns of similar polarity, aiding volatile compound identification and characterization.^31^ To test for statistically significant differences between morphotypes A and B regarding the relative area per class, the nonparametric Kruskal–Walli’s test was applied (α = 0.05). All scripts for data processing, summarization, statistical testing, and plot generation were documented in a custom R script, ensuring full reproducibility and traceability of the analyses.

## Results

Monthly precipitation data (2004–2023) were decomposed into observed, trend, seasonal, and residual components, revealing a decline from 2004 to 2012, followed by an increase until 2017, and subsequent stabilization. Seasonal patterns consistently peaked in March and April and reached their lowest in September and October, while the residual component exhibited low-amplitude variations (Figure 1).

**Figure 1:**
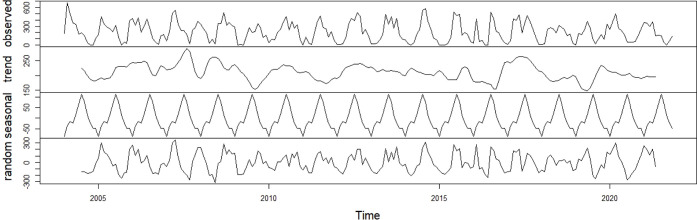
Decomposition of additive monthly rainfall time series (2004–2023).

The model chosen by “Auto.arima­()” was SARIMA­(2,0,2)­(1,0,1)[12], with a nonzero mean. The coefficients were statistically significant and exhibited low standard errors, particularly for nonseasonal terms. The residual variance (σ^2^ = 12.172), log-likelihood (−1313.740), AIC (2643.480), AICc (2644.180), and BIC (2670.440) indicated a satisfactory model fit. Residual autocorrelation at lag 1 (ACF1 = 0.024) was low, confirming approximate residual independence. The augmented Dickey–Fuller test yielded a test statistic of −10.51 (*p* = 0.010), indicating stationarity. The residual analysis showed oscillations around zero with no remaining trends or seasonality, and the residual histogram indicated an approximately normal distribution with mild outliers. The autocorrelation function (ACF) of the residual revealed no significant autocorrelation for up to 36 lags, and the Ljung–Box test (Q* = 16.816; d*f* = 18; *p* = 0.535) confirmed residual independence (Figure 2).

**Figure 2:**
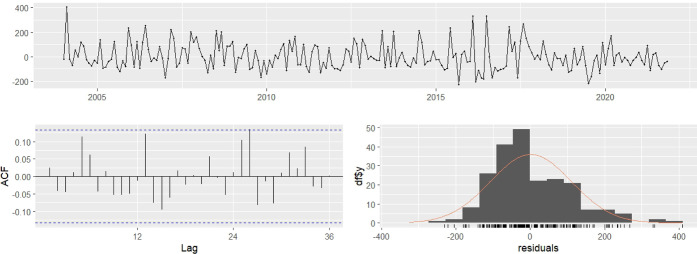
Residuals from the SARIMA (2,0,2)­(1,0,1)­[s = 12] model with time series, ACF, and frequency distribution.

Using the validated model, monthly precipitation forecasts for 2024 were generated with 95% confidence intervals, again highlighting peaks in March–April and troughs in September–October (Figure 3). Based on these forecasts, sampling dates were established for March 15 (peak rainy season), June 15 (transition from rainy to dry season), September 15 (peak dry season), and December 15 (transition from dry to rainy season).

**Figure 3:**
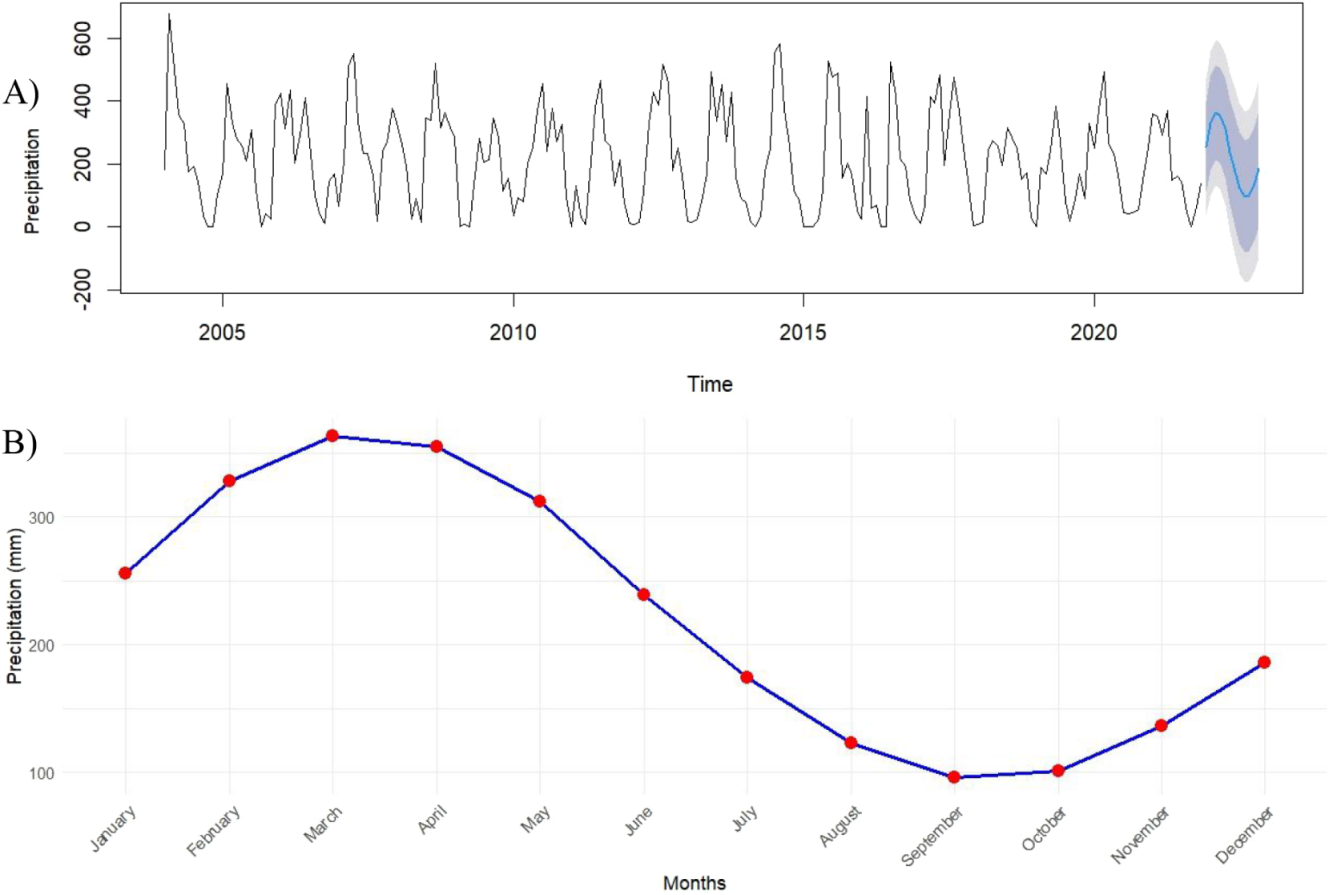
Monthly rainfall forecast via the SARIMA model (A) and seasonal variation in rainfall forecast for 2024 (B).

Overlaying the ^1^H NMR spectra confirmed the efficacy of preprocessing (baseline correction and automatic alignment), as chemical shift drifts >0.05 ppm were eliminated, and buckets became comparable. The low-intensity noise and residual solvent signals were suppressed, resulting in flat baselines with SNR 3 during bucketing. In the aliphatic region (0.8–1.5 ppm), peaks corresponding to methyl and methylene groups from saturated terpenoid chains suggested seasonal fluctuations in total monoterpene and sesquiterpene content. The 1.5–3.0 ppm range, assigned to methine and methylene protons in diverse molecular environments, indicated coexisting oxygenated terpenoid structures. Between 3.5–4.5 ppm, and peaks attributable to α-hydrogens bound to heteroatoms (−OCH– or −CH_2_– adjacent to oxygen) signified monoterpenic alcohols and ethers. In the 5.0–7.0 ppm range, olfactory and aromatic signals were discrete, reaffirming that most metabolites were aliphatic, with minor aromatic or vinylic components that varied across the sampling month (Figure 4).

**Figure 4:**
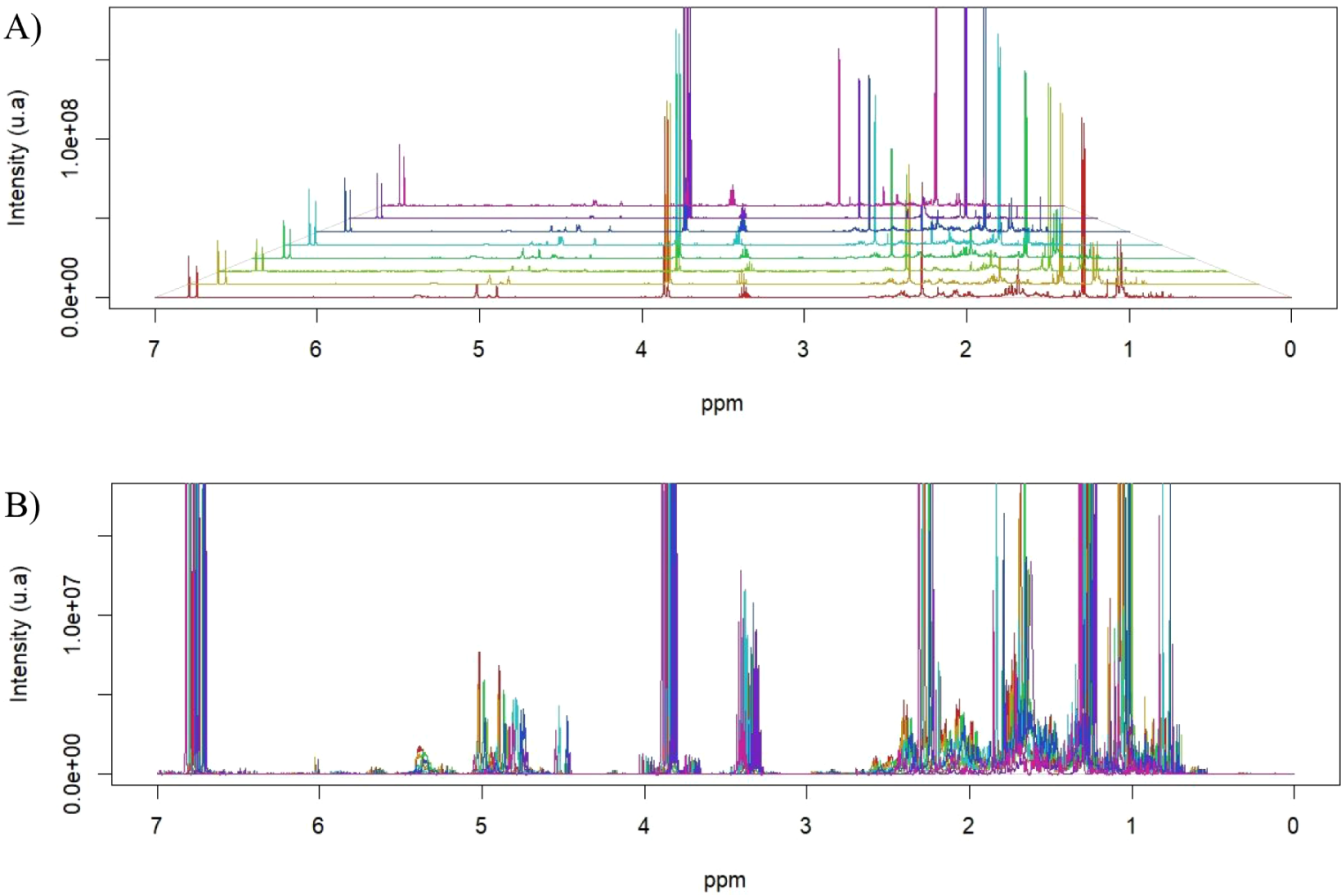
^1^H NMR spectra of the essential oils from *Ayapana triplinervis* Morphotypes A (“Japana-branca”) and B (“Japana-roxa”) collected in Macapá, Brazil, during four seasonal periods (March, June, September, and December 2024). Spectra were acquired at 500 MHz in CDCl_3_ with TMS as an internal standard.

Comparison between morphotypes revealed that Morphotype A displayed higher relative intensity in the aliphatic region and at 4.2 ppm, with more defined aromatic peaks at 6.3 and 7.0 ppm, indicating accumulation of aliphatic and aromatic compounds. In contrast, Morphotype B exhibited lower intensity in the aliphatic region and greater dispersion of polar signals at 3.7–4.0 ppm, with reduced or absent intense aromatic peaks, suggesting preferential accumulation of polar metabolites.^32^

The qualitative analysis of the chemical profiles showed that oxygenated compounds (38.40 ± 7.99% for morphotype A and 37.46 ± 1.80% for B) and aliphatic compounds were the most abundant (34.76 ± 13.66% for A and 37.53 ± 3.04% for B), followed by the allylic/methylenic, aromatic, and vinylic groups. In the seasonal profile, morphotype A exhibited the highest proportion of oxygenated compounds in March (50.20%), which gradually decreased until December (34.12%), while the aliphatic compounds increased from 14.28% in March to about 40.74% from June to December.

The B morphotype, on the other hand, maintained relatively stable fractions of oxygenated compounds (40.41–34.56%) and showed a higher aliphatic content in March (40.41%), which decreased in September (34.56%). The allylic/methylene and aromatic groups fluctuated in a complementary manner throughout the year, with an allylic peak in March (24.77% in A; 12.73% in B) and a moderate increase from September to December, while the vinyl compounds remained equal to or below 4.87% in all seasons (Figure 5).

**Figure 5:**
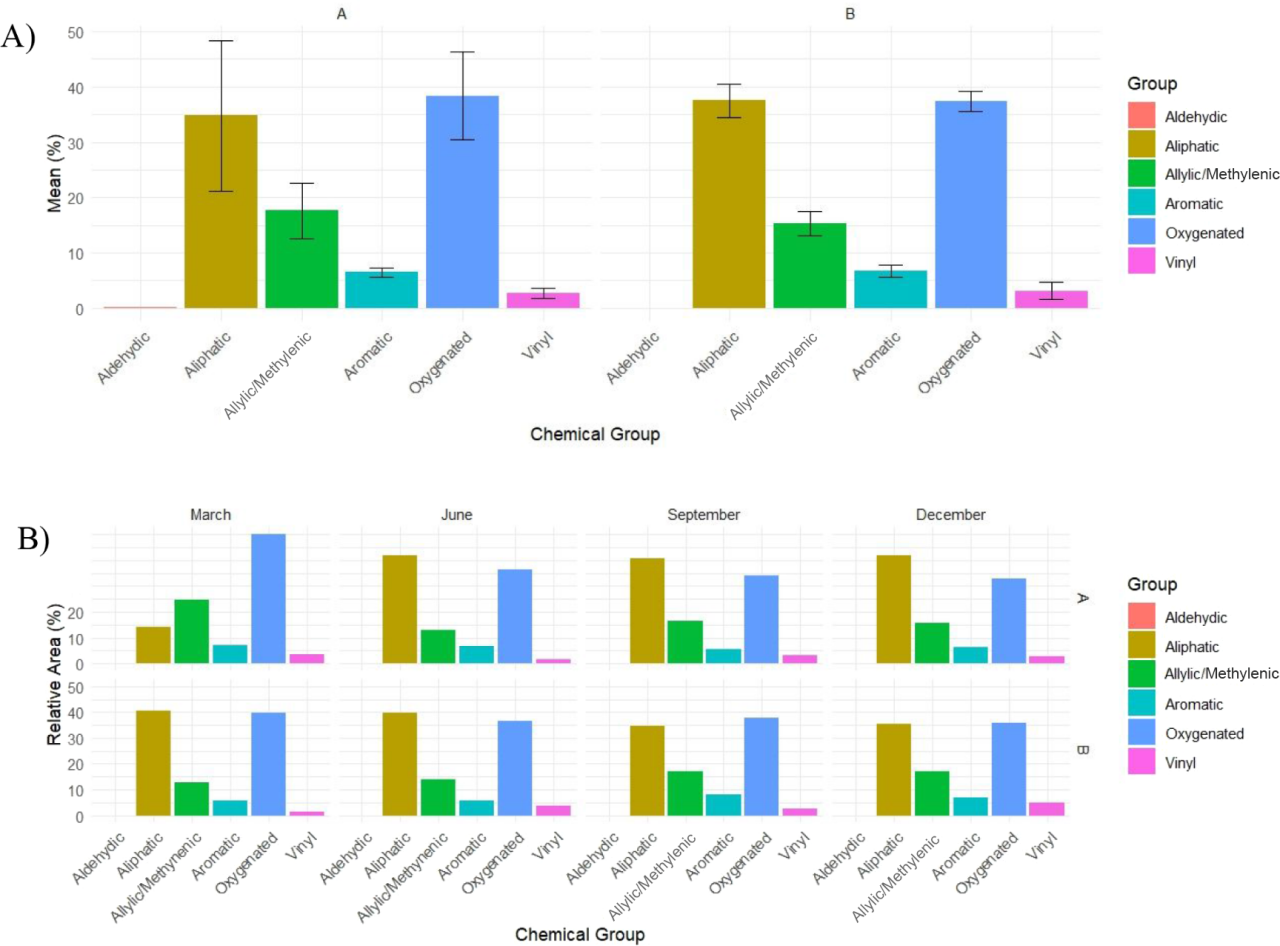
Seasonal variation in chemical group composition (%) of *A. triplinervis* essential oils determined by ^1^H NMR across four collection periods in 2024. (A) Temporal variation of chemical groups (mean ± standard deviation) for Morphotypes A and B. (B) Comparative distribution by morphotype and sampling period.

The PCA revealed two principal components, explaining 70.5% of the total variability in the essential oils of *A. triplinervis*. PC1 (50.5%) represented an oxygenation gradient, separating samples with higher levels of oxygenated compounds, while PC2 (20.0%) reflected aromaticity and unsaturation, highlighted by the accumulation of vinylic and aromatic compounds. Thus, Morphotype A exhibited a more oxidized chemical profile, whereas Morphotype B showed greater metabolic plasticity influenced by rainfall (Figure 6).

**Figure 6:**
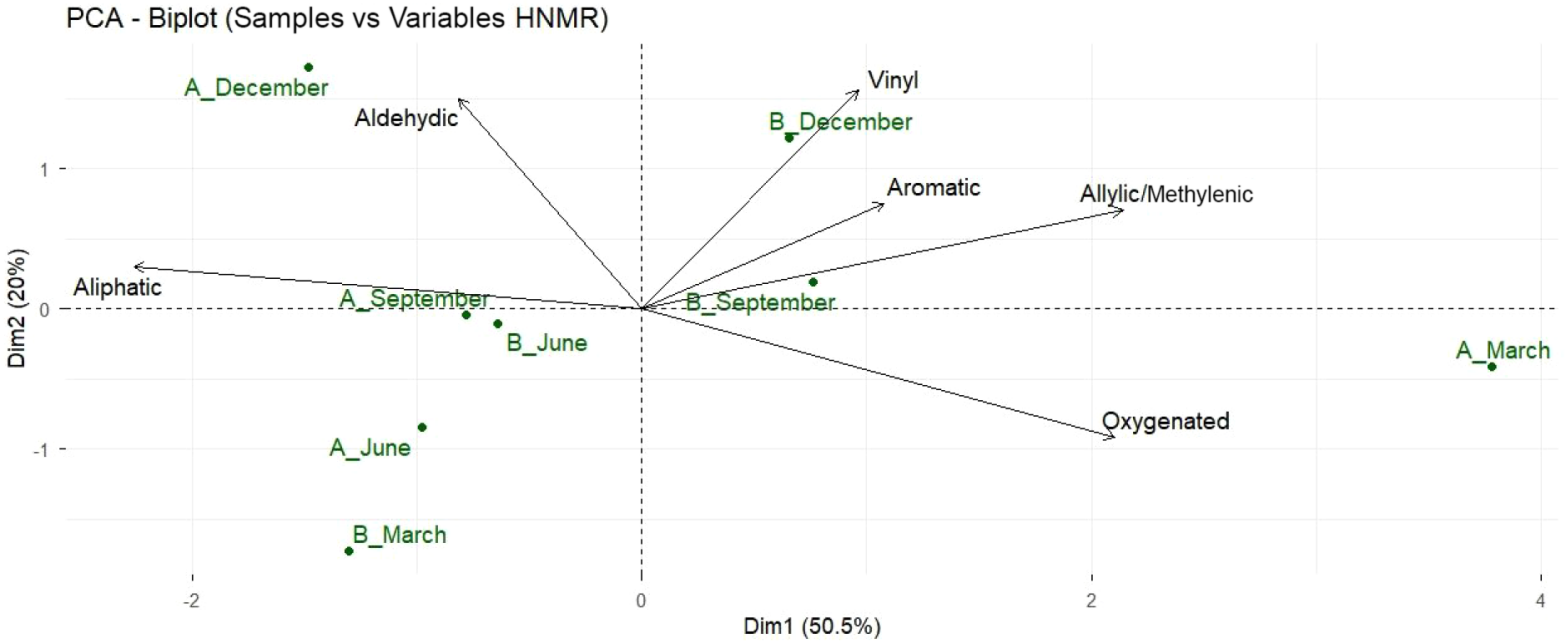
Principal Component Analysis (PCA) biplot of ^1^H NMR spectral data from *A. triplinervis* essential oils, showing sample distribution by morphotype (A, B) and season (March, June, September, December). PC1 (50.5%) represents an oxygenation gradient distinguishing highly oxygenated from aliphatic-dominant profiles; PC2 (20.0%) reflects aromaticity and unsaturation.

GC–MS analysis revealed that the number of detected compounds ranged from 18 to 23 per period–morphotype combination (March, June, September, and December; A, B). THDE dominated all groups except Morphotype A in December. In March and June, THDE was predominant in both morphotypes. In September, the relative abundances were 65.22% in B and 38.67% in A. In December, Morphotype B maintained THDE as predominant (77.11%), while in Morphotype A the main peak was cis-Caryophyllene (36.76%).

When aggregated by metabolite class regardless of morphotype, rainfall influenced secondary metabolite biosynthesis; sesquiterpenes decreased from March to June and increased in September and December (Figure 7A). In the class distribution by period and morphotype, oxygenated phenylpropanoid was predominant in all months for both morphotypes, followed by sesquiterpene. In Morphotype B, sesquiterpene increased progressively through December, and oxygenated sesquiterpene fractions peaked in September (Figure 7B).

**Figure 7:**
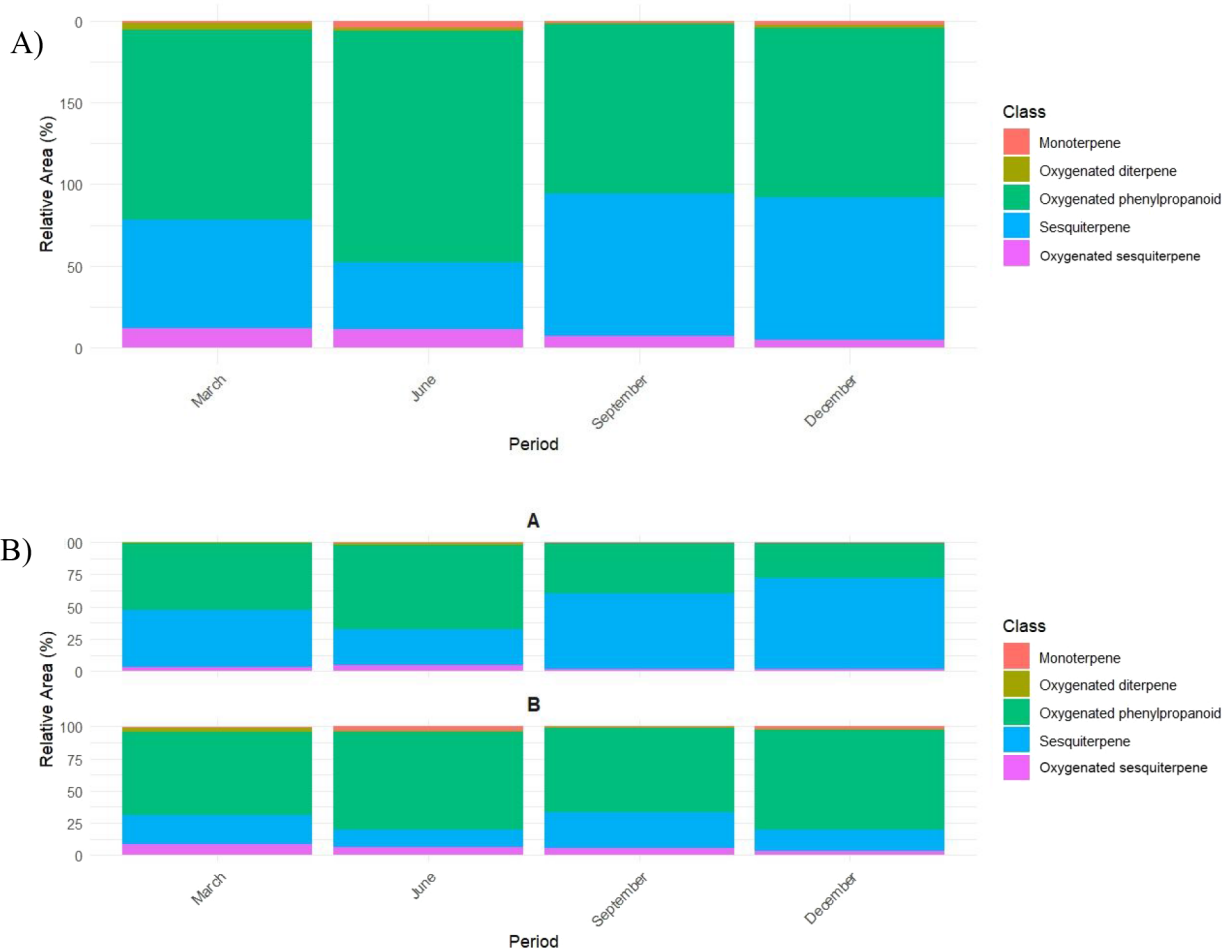
(A) Aggregated seasonal distributions of compound classes in AtEO and (B) distribution of chemical classes by period and morphotype.

The Kruskal–Walli’s test indicated a significant difference in Area (%) between the two morphotypes (χ^2^ = 11.645; d*f* = 1; *p* < 0.001). In Dunn’s posthoc test with Bonferroni correction, it was observed that morphotypes A and B differ from each other in a statistically significant way (adjusted *p* < 0.0011), with morphotype B exhibiting higher median area values than morphotype A.

In Morphotype B, THDE varied from 63.6% (March) to 77.1% (December), signifying stable production with intensification during seasonal transitions. In Morphotype A, this compound varied from 51.8% (March) to 38.7% (September), with cis-Caryophyllene predominating in December (36.8%) (Figure 8A). The Kruskal–Walli’s test showed significant differences between morphotypes for sesquiterpene (*p* = 0.008) and monoterpenes (*p* = 0.017), but not for oxygenated sesquiterpene (*p* = 0.131), oxygenated diterpene (0.100) and oxygenated phenylpropanoid (*p* = 0.520), indicating similar proportions of the latter classes throughout the year. Mean relative areas revealed that oxygenated phenylpropanoid accounted for the largest difference, averaging 40.40% in B and 30.50% in A, reflecting systematic distinctions between morphotypes in each class (Figure 8B).

**Figure 8:**
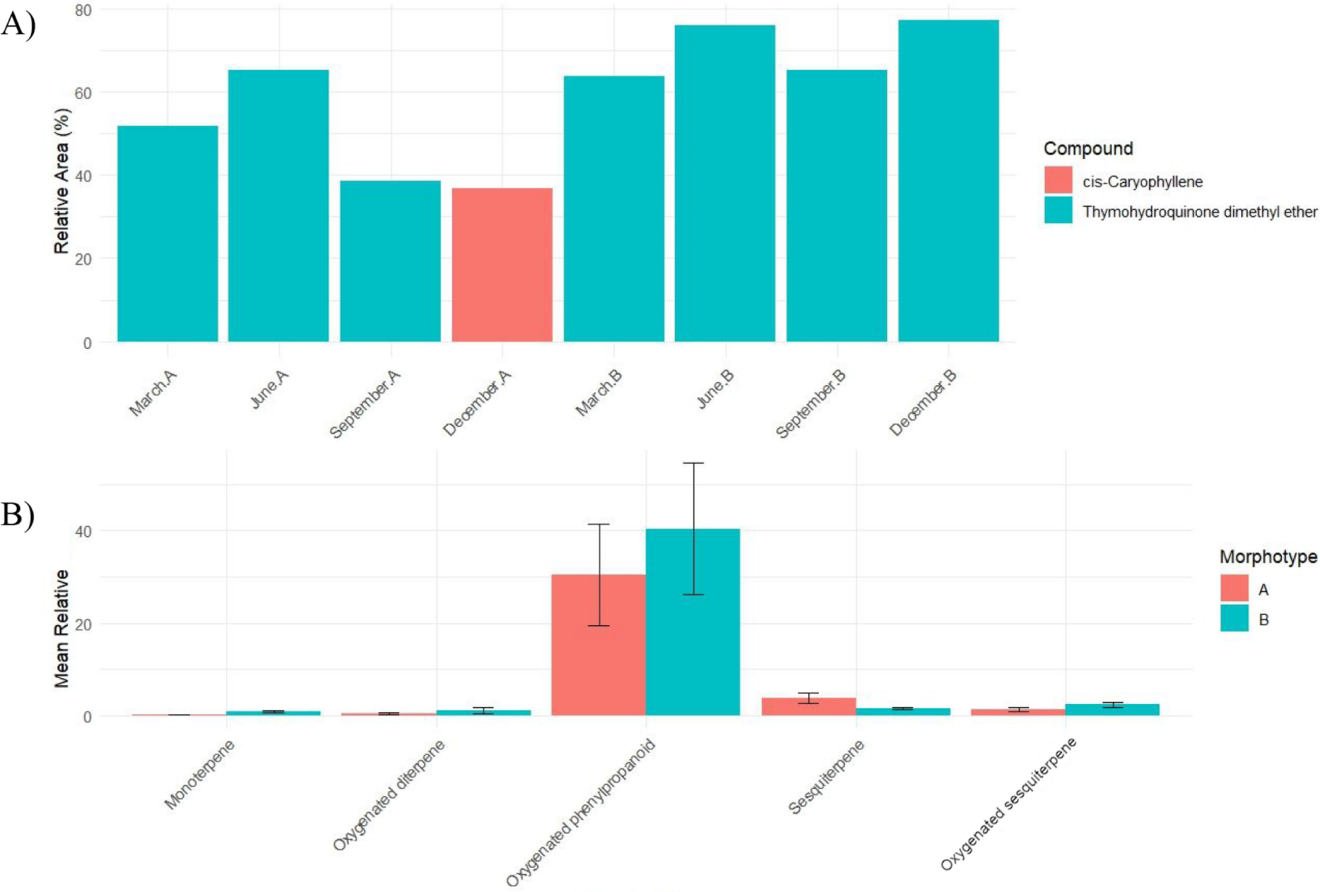
(A) Majority of compounds by period and morphotype and (B) mean and standard error of relative area by chemical class and morphotype.

The effects of the interactions were assessed using the ANOVA of ranks (Scheirer–Ray–Hare) on the area (%) values. A significant effect was identified for morphotype (*H*_1_ = 13.28; *p* < 0.001) and compound class (*H*_4_ = 3.83; *p* = 0.005), whereas the interaction between Morphotype and Class was not significant (*H*_4_ = 0.77; *p* = 0.540), indicating the preservation of the relative abundance pattern among classes in both morphotypes. Dunn’s pairwise comparisons revealed a significant contrast solely between monoterpenes and oxygenated phenylpropanoids (adjusted *p* = 0.007), with the latter being more abundant.

The ^1^H NMR and GC–MS analyses showed agreement in characterizing the seasonal and morphotype-specific variations of AtEO. ^1^H NMR indicated a predominance of oxygenated and aliphatic groups, with Morphotype A exhibiting greater seasonal variation and Morphotype B maintaining chemical stability. PCA revealed an oxygenation gradient separating the morphotypes. GC–MS confirmed THDE as the major compound, except in December for Morphotype A, when cis-caryophyllene prevailed. Thus, Morphotype B displayed a stable phenylpropanoid profile, whereas Morphotype A showed higher metabolic plasticity with an increase in sesquiterpenes during the dry period.

## Discussion

The SARIMA model forecasted a gradual increase in precipitation during the first months, followed by a reduction in the subsequent months, aligning with the region’s typical seasonal patterns.^33^ The agreement between the predicted and historical patterns indicated that the model was well-calibrated and accurately captured seasonal oscillations. Macapá, located in northern Brazil and straddling the equator, exhibits an equatorial humid climate, with constant sunlight, average temperatures of approximately 27 °C, and relative humidity above 80% throughout the year.^34^

Specifically, the SARIMA (2,0,2)­(1,0,1)[12] model captures both the seasonal structure and temporal dependencies of the monthly precipitation series. Its coefficients, low residual error, and the absence of significant residual autocorrelation confirm the suitability of the model for forecasting and inferential analysis. The highest precipitation is expected in March and April, which corresponds to the peak of the wet season in many tropical and subtropical regions. Conversely, September and October are the driest months, marking the height of the dry season.^35^

The literature further indicates that the lowest precipitation occurs in the September–October–November quarter, with averages below 60 mm, signaling the beginning of the Amazonian summer, which extends through much of the austral spring. Although rainfall significantly decreased during this period, it did not constitute a true dry season, as precipitation never fully ceased. The Amazonian winter typically begins in December, near the southern hemisphere’s summer solstice, when monthly rainfall surpasses 60 mm and intensifies until it peaks in March, known as the Equinox of Waters, with averages around 407.7 mm. This seasonal pattern is directly linked to the Intertropical Convergence Zone, the main meteorological system regulating the region’s rainfall regime.^36^

SARIMA models have been successfully applied in other contexts: the monthly rainfall time series in Enugu, Nigeria;^37^ to study the relationship between the Southern Oscillation Index and precipitation in Queensland;^38^ to forecast confirmed COVID-19 cases in Algeria using daily data from March to August 2020, capturing weekly patterns in the spread of the virus;^39^ and integrating precipitation forecasts with the Standardized Precipitation Index drought index in four zones of Jember District, Indonesia.^40^ These examples demonstrate SARIMA’s viability for assessing seasonal influences, including essential oil composition.

The morphotypes of *A. triplinervis* show a predominance of oxygenated and aliphatic compounds, with morphotype B displaying a higher percentage of aliphatic compounds. Seasonality influences the secondary metabolites, such that during the rainiest period (March), there is an increase in oxygenated metabolites, while the period of lower rainfall (June to December) stimulates the synthesis of aliphatic hydrocarbons. Similarly to our data, the literature has shown that the composition of the essential oil of *Copaifera langsdorffii* is strongly influenced by seasonality, resulting in oils with a significantly higher content of nonoxygenated sesquiterpenes during the dry season.^41^

Oxygenated phenylpropanoids are consistently predominant in both morphotypes, followed by monoterpenes, while diterpenes and sesquiterpenes (oxygenated or not) show variations without significant differences. The absence of a statistical interaction between morphotype and class suggests that, although morphotype B accumulates more material, the hierarchy of class abundance remains unchanged, indicating an additive effect of morphological and seasonal variables. Consistent with this data, the literature suggests that the “sylvestris” and “lingua” varieties of *Casearia sylvestris* can be distinguished not only by morphoanatomical criteria but also by their seasonal composition. The “lingua” variant accumulates higher levels of germacrene D, α-muurolol, and α-cadinol, while “sylvestris” is rich in (*E*)-caryophyllene, spathulenol, β-elemene, and bicyclogermacrene.^42^

The chemical composition of AtEO varies according to geographic origin, morphotype, plant organ, and to a lesser extent, developmental stage. Almost all studies have identified the same major compound, THDE (2,5-dimethoxy-*p*-cymene). However, its proportion ranges from approximately 50% in certain Indian samples to over 90% in Reunion Island samples, 60–70% in Brazil and Vietnam, and 80–87% in specific Indian locations.^24^

In the Reunion Island (Mascarene Islands) samples, THDE content ranged between 89.9% and 92.8%. Although minor variations in other constituents were observed, this compound overwhelmingly dominated the oil, regardless of the growth stage, indicating a stronger geographic than phenological influence.^1^ In southern India (Kerala), leaf and stem extracts contain THDE (80.3 to 86.9%), β-Caryophyllene (4–6%), and β-eudesmene (6–10%). These proportions remained stable across seasons, suggesting minimal seasonal variability at the site.^43^ In Vietnam (Nghệ An province), oil from stem bark and fresh leaves contained THDE between 69.90% and 88.24%, (*E*)-Caryophyllene between 3.76% and 10.90%, and β-Selinene between 3.97% and 11.79%, indicating that while the aromatic monoterpene was dominant (70–88%), its quantity varied according to the plant part (stem vs. leaf).^44^

Brazilian samples tended to exhibit intermediate levels of THDE (approximately 60–70%), with greater sesquiterpene diversity compared to the Reunion Island samples. In Tracuateua (Pará, Brazil) leaf samples, THDE reached 63.6%, β-selinene 16.4%, and E-caryophyllene 9.4%, along with smaller amounts of other sesquiterpenes. Oxygenated monoterpenes accounted for 64.3% of the oil, while hydrocarbon sesquiterpenes accounted for 29.8%.^16^ Another study in Amapá (Brazil) reported that THDE was the main compound (69.7%), followed by β-Caryophyllene (19.7%).^45^

Samples collected in Macapá (Amapá, Brazil) revealed that the two morphotypes exhibited markedly distinct profiles: in Morphotype A β-Caryophyllene predominated at 45.93% and THDE at 32.93%. In Morphotype B, THDE was the sole major compound (84.53%), whereas other sesquiterpenes and oxygenated sesquiterpenes were present at much lower concentrations (β-Caryophyllene was nearly absent); oxygenated *p*-cymene derivatives reached 86.35%. This contrast indicates that Morphotype B represents a strongly “*p*-cymene” chemotype, whereas Morphotype A is mixed, with a high proportion of sesquiterpenes, which are linked to genetic and phylogenetic factors between the two morphotypes.^46^

In Brazilian and Vietnamese samples, β-Caryophyllene appears as the second-largest fraction (9–19.7%), followed by β-Selinene at 3.97–16.4%. In Amazonian Morphotype A, β-Caryophyllene reached 45.93%, whereas in Morphotype B, its percentage was negligible. Reunion Island and Indian samples tended to show THDE levels ≥ 80%, while Brazilian samples ranged from 60 to 70%, and Vietnam showed averages between 70% and 88%, possibly influenced by specific edaphoclimatic conditions.^1,16,18^

The biosynthetic pathway of β-caryophyllene in *A. triplinervis* has attracted attention due to its anticancer potential. Our studies agree with scientific literature, which identified 25 genes encoding the 17 enzymes responsible to produce β-caryophyllene in the leaves of *A. triplinervis*. In the context of our study, this genomic and molecular knowledge enables the development of metabolic engineering strategies and standardization of secondary metabolites to optimize the production of biologically active compounds, both in heterologous systems and in the plant itself.^47^

The climatic variables have a decisive impact on essential oil production. In *Thymus algeriensis* and *Rosmarinus officinalis* cultivated in a semiarid region of Algeria, seasonal precipitation negatively influenced the oil yield in both species.^48^ Over two years, the carvacrol proportion of *T. piperella* essential oil increased with higher temperature and longer sunlight duration, peaking at the beginning of flowering (52.9% in 2018 and 41% in 2019), whereas γ-Terpinene followed a similar trend at lower values; p-cymene behaved conversely, decreasing until flowering and then increasing. These patterns reflect Carvacrol biosynthetic pathways and are related not only to seasonal temperature and photoperiod changes, but also to water availability, demonstrating that chemotype classification vary according to developmental stage.^49^

Choosing an optimal collection period can maximize target metabolite content. In *Cordia verbenacea*, the qualitative and quantitative composition of essential oils varies throughout the year, with sabinene as the major constituent in all months.^50^ Environmental and ontogenetic fluctuations are interconnected and directly affect the quantitative composition of essential oils. In *Mentha × piperita*, essential oil composition varied over three years, with fluctuations in Menthol, Menthone, Mimonene, Menthyl Acetate, Menthofuran, and β-Caryophyllene levels in response to monthly and seasonal variations in temperature and precipitation. Menthol increased during warmer periods, while Menthone and other constituents varied with plant phenological stages.^51^

Although genetic composition determines the chemotype, annual climatic variations selectively affect the quantitative stability of essential oils and their constituents in each chemotype. Meteorological factors differentially influenced essential oil accumulation and key compounds in various chemotypes of *Thymus pulegioides* cultivated at the same site. In nonphenolic chemotypes, higher temperatures and longer sunlight increased oil yields in Geraniol and Linalool chemotypes, whereas in the phenolic carvacrol chemotype none of these factors significantly affected Carvacrol content.^52^

The Citral chemotype of *Lippia alba* proved promising for developing temperature-stable topical antimicrobial formulations, with antibacterial effects attributed mainly to the high citral content along with other constituents. This chemotype’s essential oil exhibited yields of 2.2–4.3% and significant seasonal variation in Citral proportion (Neral + Geranial), rising from 49% in the rainy season to 66% in the dry season.^53^

In the present study, data confirm that compounds classified as oxygenated phenylpropanoid dominate chemical composition throughout the seasonal cycle. This predominance reflects the major role of THDE in nearly all sampling periods and in both morphotypes, peaking at 77.11% in Morphotype B in December. Sharp fluctuations in these phenylpropanoid concentrations suggest biochemical responses that are sensitive to environmental factors, such as light intensity and temperature, especially during seasonal transitions. Literature shows that essential oil production is seasonally modulated, providing crucial insights for ecological, chemosystematic, and chemophenetic studies essential for managing and conserving this medicinal species.^54^ The phenylpropanoid biosynthesis pathway depends on both developmental stage and species, with higher enzymatic activation in the early rhizome formation stages under intense UV-B radiation.^55^

The sesquiterpene class showed the second-highest accumulation and greatest compound diversity, functioning as a broadly diversified group largely comprised of minor metabolites whose sum is significant. The cis-Caryophyllene peak in Morphotype A in December (36.76%) suggests specific adjustment of this morphotype to end-of-season environmental conditions. Moreover, the shift of Morphotype A to cis-Caryophyllene instead of THDE as the major compound in December may reflect genotypic or phenological differences between morphotypes, leading to distinct secondary profiles under the same climatic regime.^56^

Caryophyllene acts as an internal chemical signal in plants, binding to the corepressor topless to activate jasmonate-dependent genes and promote resistance responses against pathogens. Its main role in plant development is to serve as a volatile messenger that adjusts immunity and indirectly contributes to plant vigor through regulation of defense hormones.^57^

The monoterpene and oxygenated diterpene classes had quantitatively limited participation, suggesting that in this species the monoterpene biosynthetic pathway is less expressive year-round, possibly restricted to abiotic stress responses such as rapid temperature or humidity changes. Oxygenated diterpenes consistently showed low and stable levels, indicating continuous production without large relative alterations, regardless of season. The consistent predominance of THDE as the major compound in most collections reinforces the ecological and pharmacological importance of oxygenated phenylpropanoids in tropical species. Phenylpropanoids are used for ultraviolet protection, potent antimicrobial action against bacteria and fungi, and antidiabetic effects by improving insulin sensitivity, anticancer activity, neuroprotection, and cardiovascular protection.^58^

In Morphotype B, THDE remained the major compound in all months, ranging from 63.6% in March to 77.1% in December. This abundance curve indicates continuous phenylpropanoid production throughout the year, peaking in December during the summer–winter transition. This behavior implies that Morphotype B constitutively prioritizes phenylpropanoid biosynthesis, likely as an antioxidant and photoprotective mechanism. This response appears intensified during extreme environmental phases, such as the peak thermal period (September) and the onset of the driest season (December).^59^

In Morphotype A, THDE also dominated during the first three months (51.8% in March, 65.2% in June, and 38.7% in September), but consistently at lower levels than in Morphotype A. Additionally, there was a sharp decline from June to September (65.2% to 38.7%), suggesting that this morphotype swiftly redirects biosynthesis to other chemical classes during the summer peak. Notably, in December, cis-Caryophyllene emerged as the major compound (36.8%), marking the only occasion when Morphotype A did not prioritize the principal phenylpropanoid. This switch to cis-Caryophyllene may reflect genotypic or regulatory differences in Morphotype A, favoring the production of nonoxygenated sesquiterpenes when conditions become drier or herbivory pressure increases.^60^

The results obtained are consistent with previously reported patterns of seasonal variation in essential oils from other Amazonian species. *Virola surinamensis* (Belém, Brazil) exhibited both seasonal and circadian fluctuations in the balance between monoterpenes, sesquiterpenes, and phenylpropanoids, reflecting metabolic adaptation mechanisms to climatic oscillations between the Amazonian wet and dry periods. Monoterpenes peaked in June, during the transition from the rainy to the dry season, whereas sesquiterpenes were most abundant in October. The phenylpropanoid elemicin showed a progressive decline throughout this transition, reaching its lowest concentration in December.^61^

Complementarily, another study demonstrated that rainfall regimes modulate both the chemical composition and biological activity of Amazonian aromatic plants, promoting an increase in sesquiterpenes during the dry season and enhancing biological potential. The essential oils of *Eugenia uniflora* contained curzerene as the major constituent in both rainy (48.1%) and dry (49.44%) seasons; *Lantana camara* was dominated by germacrene D (26.69% and 32.90%); *Ocimum basilicum* by methyl chavicol (35.30% and 41.80%); and *Plectranthus neochilus* by caryophyllene (29.84% and 41.82%). Despite the preservation of major compounds, qualitative and quantitative shifts were observed in composition and bioactivity against *Aedes aegypti*. These parallels support the hypothesis that the phenylpropanoid stability of Morphotype B and the sesquiterpenic plasticity of Morphotype A represent complementary metabolic strategies of ecological adaptation to hydric and light gradients.^62^

The SARIMA forecasts aligned with ^1^H NMR and GC–MS data, showing that rainfall regulates the balance between oxygenated phenylpropanoids and sesquiterpenes. Morphotype B maintained stable production of THDE, indicating a constitutive and protective metabolism, while Morphotype A shifted toward cis-caryophyllene under transition conditions. Differences between morphotypes indicate relevant intraspecific genetic diversity for conservation or bioprospecting programs and underscore the need to consider both seasonal effects and morphotypic variation to understand secondary metabolite profiles in Amazonian species.

## Conclusion

The integration of climate forecasting and metabolomic profiling revealed that *A. triplinervis* exhibits clear morphotype-specific and seasonal metabolic patterns. Morphotype B maintains a stable, phenylpropanoid-dominated profile, while Morphotype A displays greater metabolic flexibility, shifting toward sesquiterpenes during dry periods. These metabolic variations suggest differentiated adaptive strategies with ecological implications and potential biotechnological applications for each morphotype.

## Supplementary Material



## Data Availability

The data supporting the findings of this study are available within the article and its Supporting Information.

## Notes

Table S1: qualitative and quantitative characterization of chemical groups from ^1^H NMR spectra of *Ayapana triplinervis* essential oils; Figure S1: GC–MS chromatograms of the major volatile compounds identified in *Ayapana triplinervis* essential oils across morphotypes and seasonal periods; Table S2: volatile compound profile of *Ayapana triplinervis* essential oils identified by GC–MS across seasonal periods and morphotypes (PDF)
