# Supplementary material for: Integrating SARIMA Forecasting and Metabolomics to Decode Seasonal Chemotype Variation in Ayapana triplinervis
Source: ACS Omega. 2025 Nov 27;10(48):59963–75. doi: 10.1021/acsomega.5c10960 (PMC12772415; doi:10.1021/acsomega.5c10960)
Supplement: Supplementary file 1 [file ao5c10960_si_001.pdf]

INTEGRATING SARIMA FORECASTING AND METABOLOMICS TO DECODE SEASONAL CHEMOTYPE VARIATION IN *Ayapana triplinervis*

Jonathan Lopes de Matos [1], Lucas de Sena Pantoja [2], Kryssia Jarina Tavares Monteiro [1], Lethicia Barreto Brandão [1], Victor Hugo de Souza Marinho [2], Irlon Maciel Ferreira [2], Fábio Rodrigues de Oliveira [1], Ryan da Silva Ramos [2], Alex Bruno Lobato Rodrigues [2,]*

[1]*Department of Biological and Health Sciences, Federal University of Amapá, 68902-280, Macapá, AP, Brazil.*

[2]*Department of Exact and Technological Sciences, Federal University of Amapá, 68902-280, Macapá, AP, Brazil.*

**Corresponding author*

E-mail: alex.rodrigues@unifap.br

Supporting Information

Table S1. Qualitative and Quantitative Characterization of Chemical Groups from ^{1}H NMR Spectra of *Ayapana triplinervis* Essential Oils. ^{1}H NMR signals were integrated and assigned based on chemical shift (δ), absolute intensity, and relative area (%) for each morphotype and seasonal collection period. Analyses were performed using a Bruker Avance III 500 MHz spectrometer with deuterated chloroform as solvent and TMS as internal reference. Processing and Analysis: Global baseline correction and automatic alignment were applied to minimize chemical shift drifts greater than 0.05 ppm, and a least-squares fitting algorithm was used for fine-tuning peaks positions, ensuring consistency among replicates. The Adaptive Intelligent Binning method was employed for spectral segmentation, dividing each spectrum into buckets into 0.5 ppm with a signal-to-noise ratio (SNR) threshold of 3. Normalized intensity values for each bucket were exported for statistical analysis in RStudio. The resulting intensity matrix was subjected to Principal Component Analysis (PCA), with scores and loadings exported for custom plotting, enabling visualization of seasonal patterns and morphotype differences and the number of principal components retained was determined according to Kaiser's criterion

Morphotype	Month	Peak	δ (ppm)	Intensity (abs)	Area (%)	Group
A	December	1	10.0775	1096688.0	0.14020907	Aldehydic
A	December	2	7.9491	570695.8	0.07296216	Aromatic
A	December	3	7.9352	633025.0	0.08093081	Aromatic
A	December	4	7.9324	585596.3	0.07486715	Aromatic
A	December	5	7.6991	133354.8	0.01704910	Aromatic
A	December	6	7.6843	330563.5	0.04226179	Aromatic
A	December	7	7.6695	270460.3	0.03457773	Aromatic
A	December	8	7.5869	576858.3	0.07375002	Aromatic
A	December	9	7.5727	303064.0	0.03874604	Aromatic
A	December	10	7.2831	4736111.5	0.60550110	Solvent
A	December	11	6.7900	25834969.8	3.30294222	Aromatic
A	December	12	6.7419	19951098.0	2.55070258	Aromatic
A	December	13	5.3966	1528486.0	0.19541346	Vinyl
A	December	14	5.3876	1718297.3	0.21968040	Vinyl
A	December	15	5.3775	1694108.0	0.21658786	Vinyl
A	December	16	5.3682	1536410.8	0.19642663	Vinyl
A	December	17	5.0172	7669311.5	0.98050406	Vinyl
A	December	18	4.8948	6801655.5	0.86957621	Vinyl
A	December	19	4.0027	848864.5	0.10852540	Oxygenated
A	December	20	3.9808	803676.5	0.10274822	Oxygenated
A	December	21	3.9522	361045.0	0.04615878	Oxygenated
A	December	22	3.8625	113546652.8	14.51668174	Oxygenated
A	December	23	3.8405	112049322.0	14.32525141	Oxygenated
A	December	24	3.7168	726053.5	0.09282429	Oxygenated
A	December	25	3.6948	759035.3	0.09704093	Oxygenated
A	December	26	3.3986	2394559.3	0.30613896	Oxygenated
A	December	27	3.3847	6124898.3	0.78305433	Oxygenated
A	December	28	3.3709	8288260.3	1.05963525	Oxygenated

A	December	29	3.3570	6261264.0	0.80048839	Oxygenated
A	December	30	3.3432	2471319.3	0.31595255	Oxygenated
A	December	31	2.4050	4575089.3	0.58491477	Allylic/Methynenic
A	December	32	2.3860	4337060.8	0.55448337	Solvent
A	December	33	2.2764	72499365.5	9.26887927	Allylic/Methynenic
A	December	34	1.7401	7135826.0	0.91229915	Allylic/Methynenic
A	December	35	1.7231	8851340.8	1.13162382	Allylic/Methynenic
A	December	36	1.7027	5987705.3	0.76551452	Allylic/Methynenic
A	December	37	1.6840	23745016.5	3.03574645	Allylic/Methynenic
A	December	38	1.2898	113428347.8	14.50155672	Aliphatic
A	December	39	1.2759	109544848.8	14.00506019	Aliphatic
A	December	40	1.1371	11009085.5	1.40748658	Aliphatic
A	December	41	1.0741	35003055.5	4.47506117	Aliphatic
A	December	42	1.0505	36387429.0	4.65205018	Aliphatic
A	December	43	0.9134	2807230.5	0.35889805	Aliphatic
A	December	44	0.8795	2110799.3	0.26986089	Aliphatic
A	December	45	0.8662	3385668.5	0.43285003	Aliphatic
A	December	46	0.8327	2486150.0	0.31784863	Aliphatic
A	December	47	0.7950	3496739.3	0.44705017	Aliphatic
A	December	48	0.7549	1646031.3	0.21044136	Aliphatic
A	December	49	0.7418	1844421.3	0.23580507	Aliphatic
A	December	50	0.6278	411539.0	0.05261433	Aliphatic
A	December	51	0.6149	418060.8	0.05344812	Aliphatic
A	December	52	0.5930	463977.8	0.05931850	Aliphatic
A	June	1	7.2829	3806586.5	0.53160653	Solvent
A	June	2	6.7995	27715578.8	3.87060236	Aromatic
A	June	3	6.7508	21504170.8	3.00315194	Aromatic
A	June	4	5.0262	5659035.8	0.79030921	Vinyl
A	June	5	4.9033	5026873.5	0.70202497	Vinyl

A	June	6	3.8705	116925191.8	16.32911682	Oxygenated
A	June	7	3.8480	114142215.3	15.94046192	Oxygenated
A	June	8	3.4081	2712683.5	0.37883817	Oxygenated
A	June	9	3.3943	6772574.3	0.94581975	Oxygenated
A	June	10	3.3805	8988417.8	1.25527204	Oxygenated
A	June	11	3.3666	6774169.8	0.94604257	Oxygenated
A	June	12	3.3528	2672929.0	0.37328628	Oxygenated
A	June	13	2.2857	76032194.0	10.61822999	Allylic/Methynenic
A	June	14	1.6935	17733375.3	2.47654378	Allylic/Methynenic
A	June	15	1.2993	113354186.3	15.83041021	Aliphatic
A	June	16	1.2854	108549091.3	15.15935758	Aliphatic
A	June	17	1.1457	9144161.8	1.27702237	Aliphatic
A	June	18	1.0831	26023585.3	3.63430803	Aliphatic
A	June	19	1.0593	26466918.3	3.69622143	Aliphatic
A	June	20	0.9223	4558403.5	0.63660108	Aliphatic
A	June	21	0.8730	2912263.3	0.40671036	Aliphatic
A	June	22	0.8035	4023967.3	0.56196471	Aliphatic
A	June	23	0.7626	2144540.0	0.29949443	Aliphatic
A	June	24	0.7497	2410260.5	0.33660347	Aliphatic
A	March	1	7.2836	36715900.0	7.09362866	Solvent
A	March	2	6.7461	19674943.5	3.80126166	Aromatic
A	March	3	6.7024	15030706.5	2.90398030	Aromatic
A	March	4	5.3407	804791.0	0.15548818	Vinyl
A	March	5	5.3311	794915.0	0.15358011	Vinyl
A	March	6	4.9680	3461428.5	0.66875900	Vinyl
A	March	7	4.8488	3073927.8	0.59389262	Vinyl
A	March	8	4.7497	1021460.8	0.19734947	Vinyl
A	March	9	4.7477	1280313.5	0.24736064	Vinyl
A	March	10	4.7457	1335690.0	0.25805956	Vinyl

A	March	11	4.7353	1282236.5	0.24773217	Vinyl
A	March	12	4.7316	1392694.3	0.26907296	Vinyl
A	March	13	4.7270	1231181.3	0.23786813	Vinyl
A	March	14	4.7233	1212378.5	0.23423538	Vinyl
A	March	15	4.4645	1144948.8	0.22120774	Oxygenated
A	March	16	4.4610	1155232.8	0.22319464	Oxygenated
A	March	17	3.8261	108943081.0	21.04814976	Oxygenated
A	March	18	3.8056	111282455.8	21.50012441	Oxygenated
A	March	19	3.3445	1733256.5	0.33487067	Oxygenated
A	March	20	3.3307	4493353.8	0.86813024	Oxygenated
A	March	21	3.3168	6169460.0	1.19195930	Oxygenated
A	March	22	3.3030	4650932.0	0.89857486	Oxygenated
A	March	23	3.2892	1846049.0	0.35666254	Oxygenated
A	March	24	2.3720	2328089.3	0.44979425	Allylic/Methynenic
A	March	25	2.3566	2605915.0	0.50347107	Allylic/Methynenic
A	March	26	2.3378	1959662.0	0.37861293	Allylic/Methynenic
A	March	27	2.2276	60698123.0	11.72707043	Allylic/Methynenic
A	March	28	2.0353	2573162.0	0.49714308	Allylic/Methynenic
A	March	29	2.0290	2115046.8	0.40863376	Allylic/Methynenic
A	March	30	2.0210	2461468.3	0.47556350	Allylic/Methynenic
A	March	31	2.0145	2347099.8	0.45346714	Allylic/Methynenic
A	March	32	1.7785	4469364.8	0.86349549	Allylic/Methynenic
A	March	33	1.7767	5764858.8	1.11378905	Allylic/Methynenic
A	March	34	1.7748	4612326.3	0.89111610	Allylic/Methynenic
A	March	35	1.6930	4631169.5	0.89475668	Allylic/Methynenic
A	March	36	1.6902	5100913.3	0.98551266	Allylic/Methynenic
A	March	37	1.6721	4584170.0	0.88567622	Allylic/Methynenic
A	March	38	1.6386	12892936.5	2.49095634	Allylic/Methynenic
A	March	39	1.2806	11450929.8	2.21235605	Aliphatic

A	March	40	1.1356	3002414.8	0.58007608	Aliphatic
A	March	41	1.0235	17000782.3	3.28460520	Aliphatic
A	March	42	1.0011	21449045.8	4.14402386	Aliphatic
A	March	43	0.8199	3491048.5	0.67448167	Aliphatic
A	March	44	0.7515	7363343.3	1.42262134	Aliphatic
A	March	45	0.7084	2468965.0	0.47701189	Aliphatic
A	March	46	0.6954	2487651.8	0.48062223	Aliphatic
A	September	1	7.2833	7590437.0	0.98290655	Solvent
A	September	2	6.7652	23392489.8	3.02915778	Aromatic
A	September	3	6.7197	17928153.0	2.32156580	Aromatic
A	September	4	4.9893	5903251.0	0.76442820	Vinyl
A	September	5	4.9188	1374448.3	0.17798108	Vinyl
A	September	6	4.8685	5253617.0	0.68030530	Vinyl
A	September	7	4.7716	1419191.5	0.18377501	Vinyl
A	September	8	4.7695	1702222.8	0.22042550	Vinyl
A	September	9	4.7675	1769081.0	0.22908316	Vinyl
A	September	10	4.7560	1782707.5	0.23084769	Vinyl
A	September	11	4.7523	1891008.5	0.24487189	Vinyl
A	September	12	4.7476	1595518.8	0.20660811	Vinyl
A	September	13	4.7439	1549525.8	0.20065235	Vinyl
A	September	14	4.4866	1543235.5	0.19983781	Oxygenated
A	September	15	4.4832	1525695.3	0.19756647	Oxygenated
A	September	16	3.8416	115423844.3	14.94655080	Oxygenated
A	September	17	3.8204	116066588.3	15.02978149	Oxygenated
A	September	18	3.3677	2139123.3	0.27700095	Oxygenated
A	September	19	3.3538	5486985.8	0.71052486	Oxygenated
A	September	20	3.3400	7440596.8	0.96350332	Oxygenated
A	September	21	3.3261	5585266.5	0.72325151	Oxygenated
A	September	22	3.3123	2219646.5	0.28742813	Oxygenated

A	September	23	2.3778	3506606.0	0.45408005	Oxygenated
A	September	24	2.3600	3468531.0	0.44914961	Allylic/Methynenic
A	September	25	2.2487	68827721.8	8.91269084	Allylic/Methynenic
A	September	26	2.0548	3637005.0	0.47096577	Allylic/Methynenic
A	September	27	2.0492	3604994.3	0.46682061	Allylic/Methynenic
A	September	28	2.0398	3714710.0	0.48102801	Allylic/Methynenic
A	September	29	2.0341	3856334.3	0.49936732	Allylic/Methynenic
A	September	30	1.7980	6197457.5	0.80252580	Allylic/Methynenic
A	September	31	1.7961	7676866.0	0.99409848	Allylic/Methynenic
A	September	32	1.7942	6075891.0	0.78678382	Allylic/Methynenic
A	September	33	1.6594	19502422.3	2.52542225	Allylic/Methynenic
A	September	34	1.2606	117520683.8	15.21807631	Aliphatic
A	September	35	1.2467	116587073.0	15.09718049	Aliphatic
A	September	36	1.1096	9083793.5	1.17628538	Aliphatic
A	September	37	1.0453	27932300.0	3.61703029	Aliphatic
A	September	38	1.0222	30899097.3	4.00120902	Aliphatic
A	September	39	0.7726	9569896.5	1.23923220	Aliphatic
B	December	1	7.2828	3980825.3	0.44805044	Solvent
B	December	2	6.8035	34939172.8	3.93247904	Aromatic
B	December	3	6.7550	27551098.0	3.10093534	Aromatic
B	December	4	5.0285	1942696.8	0.21865470	Vinyl
B	December	5	4.9061	1923310.0	0.21647268	Vinyl
B	December	6	4.8139	2578171.5	0.29017875	Vinyl
B	December	7	4.8120	3819041.5	0.42984133	Vinyl
B	December	8	4.8100	4572481.0	0.51464257	Vinyl
B	December	9	4.8080	4592465.5	0.51689187	Vinyl
B	December	10	4.7978	1828720.0	0.20582637	Vinyl
B	December	11	4.7946	4370136.5	0.49186826	Vinyl
B	December	12	4.7909	4745765.8	0.53414614	Vinyl

B	December	13	4.7866	4568409.3	0.51418429	Vinyl
B	December	14	4.7829	4272233.5	0.48084907	Vinyl
B	December	15	4.7795	2842741.3	0.31995664	Vinyl
B	December	16	4.7764	1043814.5	0.11748357	Vinyl
B	December	17	4.5306	1720943.5	0.19369589	Oxygenated
B	December	18	4.5272	4233487.8	0.47648815	Oxygenated
B	December	19	4.5237	4184470.0	0.47097110	Oxygenated
B	December	20	4.5202	1691780.3	0.19041351	Oxygenated
B	December	21	3.8750	135200896.5	15.21715170	Oxygenated
B	December	22	3.8523	132752899.0	14.94162431	Oxygenated
B	December	23	3.4107	3375982.8	0.37997412	Oxygenated
B	December	24	3.3969	8557521.5	0.96316745	Oxygenated
B	December	25	3.3830	11543935.0	1.29929471	Oxygenated
B	December	26	3.3692	8707603.3	0.98005947	Oxygenated
B	December	27	3.3553	3490162.5	0.39282530	Oxygenated
B	December	28	2.2890	94007836.8	10.58078422	Allylic/Methynenic
B	December	29	2.1888	6946513.3	0.78184501	Allylic/Methynenic
B	December	30	1.8355	15304269.8	1.72252848	Allylic/Methynenic
B	December	31	1.8337	18229214.8	2.05173733	Allylic/Methynenic
B	December	32	1.6965	9615961.8	1.08229718	Allylic/Methynenic
B	December	33	1.6902	7346401.8	0.82685332	Allylic/Methynenic
B	December	34	1.3697	4365108.5	0.49130235	Aliphatic
B	December	35	1.3459	6761655.8	0.76103890	Aliphatic
B	December	36	1.3023	132732162.3	14.93929035	Aliphatic
B	December	37	1.2884	124461399.0	14.00839815	Aliphatic
B	December	38	1.0852	9525085.0	1.07206880	Aliphatic
B	December	39	1.0613	9989626.0	1.12435389	Aliphatic
B	December	40	0.8111	24161024.3	2.71937525	Aliphatic
B	June	1	7.2834	14696424.0	1.39716532	Solvent

B	June	2	6.7591	33726591.0	3.20633261	Aromatic
B	June	3	6.7142	25915913.5	2.46378409	Aromatic
B	June	4	6.0099	870439.5	0.08275128	Vinyl
B	June	5	5.3541	873496.5	0.08304190	Vinyl
B	June	6	4.9818	3546019.0	0.33711431	Vinyl
B	June	7	4.8618	3235238.5	0.30756891	Vinyl
B	June	8	4.7638	3390555.0	0.32233459	Vinyl
B	June	9	4.7618	3997669.5	0.38005199	Vinyl
B	June	10	4.7598	3989802.0	0.37930404	Vinyl
B	June	11	4.7579	2751228.5	0.26155486	Vinyl
B	June	12	4.7486	3905282.0	0.37126886	Vinyl
B	June	13	4.7450	4073423.5	0.38725380	Vinyl
B	June	14	4.7404	3698625.0	0.35162231	Vinyl
B	June	15	4.7367	3562066.0	0.33863987	Vinyl
B	June	16	4.4787	3679075.0	0.34976373	Oxygenated
B	June	17	4.4753	3543653.5	0.33688942	Oxygenated
B	June	18	3.8369	170344446.5	16.19437177	Oxygenated
B	June	19	3.8158	168974270.5	16.06411135	Oxygenated
B	June	20	3.3595	3213222.5	0.30547588	Oxygenated
B	June	21	3.3456	7964325.0	0.75715553	Oxygenated
B	June	22	3.3318	10689194.5	1.01620448	Oxygenated
B	June	23	3.3179	7929067.0	0.75380361	Oxygenated
B	June	24	3.3041	3048275.5	0.28979464	Oxygenated
B	June	25	2.2416	101582079.0	9.65724440	Allylic/Methynenic
B	June	26	1.7913	14256128.5	1.35530714	Allylic/Methynenic
B	June	27	1.7894	16423800.0	1.56138417	Allylic/Methynenic
B	June	28	1.6531	14144937.5	1.34473639	Allylic/Methynenic
B	June	29	1.2529	175580231.5	16.69212940	Aliphatic
B	June	30	1.2390	166012744.0	15.78256380	Aliphatic

B	June	31	1.1022	4843872.0	0.46049910	Aliphatic
B	June	32	1.0376	17180384.0	1.63331140	Aliphatic
B	June	33	1.0146	20526989.0	1.95146773	Aliphatic
B	June	34	0.8323	3554781.0	0.33794730	Aliphatic
B	June	35	0.7650	21258149.0	2.02097794	Aliphatic
B	June	36	0.7208	2496922.5	0.23737839	Aliphatic
B	June	37	0.7078	2395051.5	0.22769368	Aliphatic
B	March	1	7.2836	63995067.0	6.79099946	Solvent
B	March	2	6.7444	28629974.0	3.03814258	Aromatic
B	March	3	6.7009	22062410.0	2.34120881	Aromatic
B	March	4	4.9660	559988.5	0.05942461	Vinyl
B	March	5	4.8447	517437.5	0.05490920	Vinyl
B	March	6	4.7476	1227922.5	0.13030412	Vinyl
B	March	7	4.7455	1532663.0	0.16264244	Vinyl
B	March	8	4.7436	1537130.0	0.16311646	Vinyl
B	March	9	4.7333	1353417.5	0.14362134	Vinyl
B	March	10	4.7295	1463057.0	0.15525602	Vinyl
B	March	11	4.7250	1374438.0	0.14585199	Vinyl
B	March	12	4.7213	1361010.0	0.14442704	Vinyl
B	March	13	4.4623	1282661.0	0.13611284	Oxygenated
B	March	14	4.4588	1306176.5	0.13860824	Oxygenated
B	March	15	3.8245	158122326.5	16.77955324	Oxygenated
B	March	16	3.8040	161386994.5	17.12599179	Oxygenated
B	March	17	3.3421	2562901.5	0.27196882	Oxygenated
B	March	18	3.3282	6612576.5	0.70171039	Oxygenated
B	March	19	3.3144	9051142.0	0.96048498	Oxygenated
B	March	20	3.3005	6821146.5	0.72384333	Oxygenated
B	March	21	3.2867	2720183.0	0.28865914	Oxygenated
B	March	22	2.2254	87792968.0	9.31637431	Allylic/Methynenic

B	March	23	1.7747	5968228.0	0.63333371	Allylic/Methynenic
B	March	24	1.7728	4652962.5	0.49376096	Allylic/Methynenic
B	March	25	1.6239	13442133.5	1.42644622	Allylic/Methynenic
B	March	26	1.2363	169305159.5	17.96624803	Aliphatic
B	March	27	1.2224	165836969.5	17.59821222	Aliphatic
B	March	28	1.1331	5290680.5	0.56143403	Aliphatic
B	March	29	1.0212	2963515.5	0.31448099	Aliphatic
B	March	30	0.9989	3483637.0	0.36967501	Aliphatic
B	March	31	0.7493	8134354.0	0.86319768	Aliphatic
B	September	1	7.2826	3481927.5	0.40223811	Solvent
B	September	2	6.8245	38470852.0	4.44421741	Aromatic
B	September	3	6.7747	30404364.5	3.51236323	Aromatic
B	September	4	5.0497	1447624.5	0.16723201	Vinyl
B	September	5	4.9266	1328476.5	0.15346784	Vinyl
B	September	6	4.8339	2398082.0	0.27703046	Vinyl
B	September	7	4.8319	2919155.5	0.33722574	Vinyl
B	September	8	4.8299	2923173.5	0.33768991	Vinyl
B	September	9	4.8154	2856508.0	0.32998860	Vinyl
B	September	10	4.8116	3170908.5	0.36630867	Vinyl
B	September	11	4.8077	3175608.0	0.36685157	Vinyl
B	September	12	4.8041	2760677.0	0.31891804	Vinyl
B	September	13	4.5490	2715512.0	0.31370050	Oxygenated
B	September	14	4.5455	2716054.0	0.31376312	Oxygenated
B	September	15	4.0330	1147562.0	0.13256829	Oxygenated
B	September	16	4.0096	1208700.0	0.13963105	Oxygenated
B	September	17	3.8930	140015585.5	16.17483549	Oxygenated
B	September	18	3.8695	135810828.5	15.68909490	Oxygenated
B	September	19	3.7471	1214508.0	0.14030200	Oxygenated
B	September	20	3.7236	1235062.5	0.14267649	Oxygenated

B	September	21	3.4337	3746286.0	0.43277725	Oxygenated
B	September	22	3.4199	9562113.0	1.10463135	Oxygenated
B	September	23	3.4061	12752656.5	1.47320829	Oxygenated
B	September	24	3.3922	9770128.5	1.12866165	Oxygenated
B	September	25	3.3783	3956620.5	0.45707544	Oxygenated
B	September	26	2.3107	98800734.0	11.41362665	Allylic/Methynenic
B	September	27	2.2041	5518236.5	0.63747594	Allylic/Methynenic
B	September	28	1.8560	9511891.5	1.09882967	Allylic/Methynenic
B	September	29	1.8540	11691192.0	1.35058613	Allylic/Methynenic
B	September	30	1.8521	9093765.0	1.05052700	Allylic/Methynenic
B	September	31	1.7159	7080763.5	0.81798169	Allylic/Methynenic
B	September	32	1.7093	4737261.5	0.54725640	Allylic/Methynenic
B	September	33	1.3247	137061026.0	15.83351981	Aliphatic
B	September	34	1.3108	129932659.5	15.01003895	Aliphatic
B	September	35	1.1065	7272581.0	0.84014077	Aliphatic
B	September	36	1.0821	7426352.0	0.85790465	Aliphatic
B	September	37	0.8314	16322953.0	1.88565493	Aliphatic

Figure S1. GC–MS chromatograms of the major volatile compounds identified in *Ayapana triplinervis* essential oils across morphotypes and seasonal periods. (A, C, D, E) Morphotype A: March, June, September, and December; (E, D, F, H) Morphotype B: March, June, September, and December, respectively. Analyses were performed using a Shimadzu GCMS-QP 5050A system with a DB-5HT capillary column under the conditions described in the methodology section.

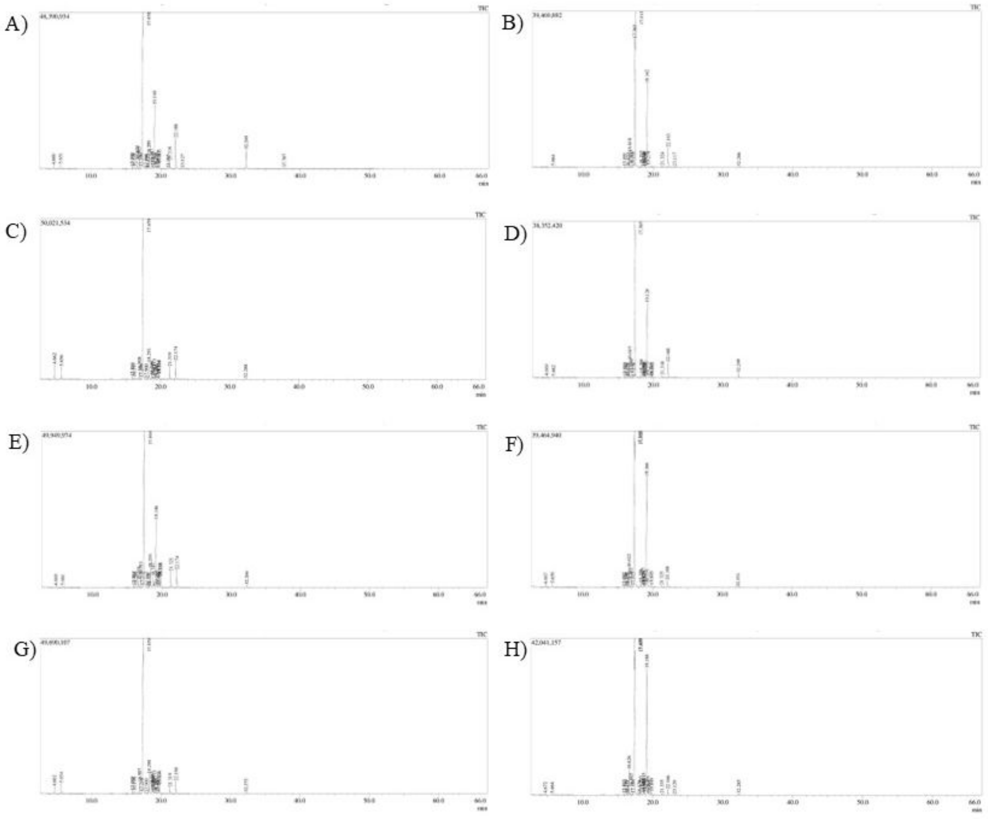

Table S2. Volatile Compound Profile of *Ayapana triplinervis* Essential Oils Identified by GC–MS across Seasonal Periods and Morphotypes. Descriptive and exploratory analysis: Raw GC-MS data were organized into a spreadsheet containing, for each peak, the sampling period (March, June, September, or December), morphotype (A or B), retention time (RT), compound name, relative abundance, LRI, and chemical classification (monoterpene, sesquiterpene, oxygenated sesquiterpene, oxygenated diterpene, and oxygenated phenylpropanoid). This spreadsheet was imported into RStudio and pre-processed. Subsequently, for each period–morphotype combination, the total number of detected compounds, the sum of normalized relative areas, and the mean and standard deviation of these areas were calculated. To assess overall chemical diversity, the number of distinct compounds per class was determined, along with the sum, mean, and standard deviation of the relative areas within each class.

Month	Morphotype	Peak	Retention Times	Compound	Area (%)	Linear Retention Index	Class
March	B	1	4.660	α-Pinene	0.44	948	Monoterpene
March	B	2	5.651	β-Pinene	0.55	943	Monoterpene
March	B	3	15.890	Dehydro Aromadendrene	0.36	1396	Sesquiterpene
March	B	4	16.198	β-maaliene	0.34	1380	Sesquiterpene
March	B	5	16.624	β-Elemene	1.6	1398	Sesquiterpene
March	B	6	16.905	α-gurjuneno	1.28	1419	Sesquiterpene
March	B	7	17.200	4-t-Butyl-1,2-dimethoxy benzene	0.32	1386	Oxygenated phenylpropanoid
March	B	8	17.458	Thymohydroquinone dimethyl ether	63.61	1423	Oxygenated phenylpropanoid
March	B	9	17.900	β-Bourbonene	0.34	1339	Sesquiterpene
March	B	10	18.125	γ-Maaliene	0.13	1398	Sesquiterpene
March	B	11	18.289	α-Caryophyllene	2.78	1579	Sesquiterpene
March	B	12	18.747	Aciphyllene	1.13	1490	Sesquiterpene
March	B	13	18.845	α-Panasinsen	1.17	1416	Sesquiterpene
March	B	14	19.140	Eudesma-4(14),11-diene	11.39	1469	Sesquiterpene
March	B	15	19.278	β-Chamigrene	0.69	1507	Sesquiterpene
March	B	16	19.475	δ-Guaiene	0.19	1490	Sesquiterpene
March	B	17	19.717	γ-Cadinene	0.74	1435	Sesquiterpene

March	B	18	19.835	γ-selinene	1.01	1461	Sesquiterpene
March	B	19	21.065	Caryophyllene oxide	0.34	1507	Oxygenated sesquiterpene
March	B	20	21.319	6-epi-shyobunol	1.57	1555	Oxygenated sesquiterpene
March	B	21	22.188	α-Cedrene epoxide	6.16	1293	Oxygenated sesquiterpene
March	B	22	23.127	Neointermedeol	0.25	1632	Oxygenated sesquiterpene
March	B	23	32.244	Phytol	3.42	2045	Oxygenated diterpene
June	B	1	4.662	α-Pinene	1.95	948	Monoterpene
June	B	2	5.656	β-Pinene	1.74	943	Monoterpene
June	B	3	15.865	Dehydro Aromadendrene	0.5	1396	Sesquiterpene
June	B	4	16.197	Calarene	0.5	1442	Sesquiterpene
June	B	5	16.908	α-gurjuneno	1.6	1419	Sesquiterpene
June	B	6	17.206	4-t-Butyl-1,2-dimethoxy benzene	0.36	1386	Oxygenated phenylpropanoid
June	B	7	17.459	Thymohydroquinone dimethyl ether	75.87	1423	Oxygenated phenylpropanoid
June	B	8	17.900	β-Bourbonene	0.35	1339	Sesquiterpene
June	B	9	18.291	α-Caryophyllene	3.84	1579	Sesquiterpene
June	B	10	18.749	Aciphyllene	0.91	1490	Sesquiterpene
June	B	11	18.845	α-Panasinsen	1.22	1416	Sesquiterpene
June	B	12	19.113	Eudesma-4(14),11-diene	1.43	1469	Sesquiterpene
June	B	13	19.295	(+)-δ-Selinene	0.42	1481	Sesquiterpene
June	B	14	19.460	δ-Guaiene	0.19	1490	Sesquiterpene
June	B	15	19.714	γ-Cadinene	1	1435	Sesquiterpene
June	B	16	19.835	γ-Gurjunene	1.17	1461	Sesquiterpene

June	B	17	21.319	Caryophyllene oxide	2.44	1507	Sesquiterpene oxigenado
June	B	18	22.174	α-Cedrene epoxide	4.17	1293	Sesquiterpene oxigenado
June	B	19	32.288	Phytol	0.35	2045	Oxygenated diterpene
September	B	1	4.669	α-Pinene	0.2	948	Monoterpene
September	B	2	5.661	β-Pinene	0.23	943	Monoterpene
September	B	3	15.894	Dehydro Aromadendrene	0.37	1396	Sesquiterpene
September	B	4	16.200	Calarene	0.5	1442	Sesquiterpene
September	B	5	16.631	β-Elemene	1.3	1398	Sesquiterpene
September	B	6	16.911	β-Maaliene	1.89	1432	Sesquiterpene
September	B	7	17.212	Aristolene	0.41	1403	Sesquiterpene
September	B	8	17.464	Thymohydroquinone dimethyl ether	65.22	1423	Oxygenated phenylpropanoid
September	B	9	17.897	β-Bourbonene	0.31	1339	Sesquiterpene
September	B	10	18.120	γ-Maaliene	0.19	1398	Sesquiterpene
September	B	11	18.293	α-Caryophyllene	3.73	1579	Sesquiterpene
September	B	12	18.747	Aciphyllene	3.4	1490	Sesquiterpene
September	B	13	19.146	Eudesma-4(14),11-diene	11.26	1469	Sesquiterpene
September	B	14	19.285	β-Chamigrene	0.78	1507	Sesquiterpene
September	B	15	19.466	δ-Guaiene	0.36	1490	Sesquiterpene
September	B	16	19.715	γ-Cadinene	1.6	1435	Sesquiterpene
September	B	17	19.838	γ-Gurjunene	1.93	1461	Sesquiterpene
September	B	18	21.321	Caryophyllene oxide	2.36	1507	Oxygenated sesquiterpene
September	B	19	22.174	α-Cedrene oxide	3.27	1293	Oxygenated sesquiterpene
September	B	20	32.266	Phytol	0.69	2045	Oxygenated diterpene

December	B	1	4.662	α-Pinene	0.9	948	Monoterpene
December	B	2	5.654	β-Pinene	1.24	943	Monoterpene
December	B	3	15.889	Aristol-1(10)-en-9-yl isovalerate	0.5	1396	Oxygenated diterpene
December	B	4	16.196	Dehydro Aromadendrene	0.63	1396	Sesquiterpene
December	B	5	16.907	α-Maaliene	2.06	1432	Sesquiterpene
December	B	6	17.216	Dimethoxydurene	0.25	1511	Oxygenated phenylpropanoid
December	B	7	17.459	Thymohydroquinone dimethyl ether	77.11	1423	Oxygenated phenylpropanoid
December	B	8	17.900	β-Bourbonene	0.29	1339	Sesquiterpene
December	B	9	18.290	α-Caryophyllene	4.11	1579	Sesquiterpene
December	B	10	18.745	Aciphyllene	1.48	1490	Sesquiterpene
December	B	11	18.845	α-Panasinsen	0.94	1416	Sesquiterpene
December	B	12	18.920	Germacrene D	0.71	1515	Sesquiterpene
December	B	13	19.111	Eudesma-4(14),11-diene	1.85	1469	Sesquiterpene
December	B	14	19.295	δ-selinene	0.41	1481	Sesquiterpene
December	B	15	19.460	δ-Guaiene	0.26	1490	Sesquiterpene
December	B	16	19.712	γ-Cadinene	1.34	1435	Sesquiterpene
December	B	17	19.834	γ-Gurjunene	1.8	1461	Sesquiterpene
December	B	18	21.319	Caryophyllene oxide	1.19	1507	Oxygenated sesquiterpene
December	B	19	22.166	α-Cedrene epoxide	2.49	1293	Oxygenated sesquiterpene
December	B	20	32.272	Phytol	0.44	2045	Oxygenated diterpene
March	A	1	5.664	Pinene	0.15	943	Monoterpene
March	A	2	15.891	Dehydro Aromadendrene	0.15	1396	Sesquiterpene
March	A	3	16.420	Germacrene B	0.17	1398	Sesquiterpene
March	A	4	16.618	β-Elemene	2.32	1398	Sesquiterpene

March	A	5	16.900	α-gurjunene	0.32	1419	Sesquiterpene
March	A	6	17.175	4-tert-Butylcatechol dimethyl ether	0.32	1386	Oxygenated phenylpropanoid
March	A	7	17.365	Caryophyllene	26.31	1494	Sesquiterpene
March	A	8	17.513	Thymohydroquinone dimethyl ether	51.79	1423	Oxygenated phenylpropanoid
March	A	9	18.297	α-Caryophyllene	0.36	1579	Sesquiterpene
March	A	10	18.655	4,5-di-epi-aristolochene	0.13	1474	Sesquiterpene
March	A	11	18.748	Aciphyllene	0.39	1490	Sesquiterpene
March	A	12	18.849	α-Panasinsene	0.1	1416	Sesquiterpene
March	A	13	19.142	Eudesma-4(14),11-diene	13.1	1469	Sesquiterpene
March	A	14	19.278	Longifolene	0.37	1398	Sesquiterpene
March	A	15	21.324	Caryophyllene oxide	0.24	1507	Oxygenated sesquiterpene
March	A	16	22.163	α-Cedrene epoxide	3.18	1293	Oxygenated sesquiterpene
March	A	17	23.117	Thunbergol	0.2	2211	Oxygenated diterpene
March	A	18	32.286	Phytol	0.39	2045	Oxygenated diterpene
June	A	1	4.669	α-Pinene	0.35	948	Monoterpene
June	A	2	5.662	β-Pinene	0.23	943	Monoterpene
June	A	3	15.888	Dehydro Aromadendrene	0.35	1396	Sesquiterpene
June	A	4	16.194	Calarene	0.28	1442	Sesquiterpene
June	A	5	16.426	Germacrene B	0.25	1570	Sesquiterpene
June	A	6	16.615	β-Elemene	3.96	1398	Sesquiterpene
June	A	7	16.899	α-Maaliene	0.99	1432	Sesquiterpene
June	A	8	17.178	4-tert-Butylcatechol, dimethyl ether	0.29	1386	Oxygenated phenylpropanoid

June	A	9	17.385	Thymohydroquinone dimethyl ether	65.24	1423	Oxygenated phenylpropanoid
June	A	10	18.266	α-Caryophyllene	0.96	1579	Sesquiterpene
June	A	11	18.640	4,5-di-epi-aristolochene	0.16	1474	Sesquiterpene
June	A	12	18.736	Aciphyllene	0.62	1490	Sesquiterpene
June	A	13	18.834	α-Panasinsene	0.2	1416	Sesquiterpene
June	A	14	19.126	Eudesma-4(14),11-diene	18.66	1469	Sesquiterpene
June	A	15	19.271	Valencene	0.66	1474	Sesquiterpene
June	A	16	19.715	γ-Muurolene	0.25	1435	Sesquiterpene
June	A	17	19.842	γ-Gurjunene	0.43	1461	Sesquiterpene
June	A	18	21.318	Caryophyllene oxide	0.65	1507	Oxygenated sesquiterpene
June	A	19	22.160	α-Cedrene oxide	4.04	1293	Oxygenated sesquiterpene
June	A	20	32.249	Phytol	1.44	2045	Oxygenated diterpene
September	A	1	4.667	α-Pinene	0.33	948	Monoterpene
September	A	2	5.659	β-Pinene	0.4	943	Monoterpene
September	A	3	15.892	Dehydro Aromadendrene	0.13	1396	Sesquiterpene
September	A	4	16.199	Calarene	0.09	1442	Sesquiterpene
September	A	5	16.429	Germacrene B	0.21	1570	Sesquiterpene
September	A	6	16.662	β-Elemene	2.74	1398	Sesquiterpene
September	A	7	16.905	α-Maaliene	0.74	1432	Sesquiterpene
September	A	8	17.175	Aristolene	0.35	1403	Sesquiterpene
September	A	9	17.385	β-Caryophyllene	33.56	1494	Sesquiterpene
September	A	10	17.509	Thymohydroquinone dimethyl ether	38.67	1423	Oxygenated phenylpropanoid
September	A	11	18.296	α-Caryophyllene	0.48	1579	Sesquiterpene
September	A	12	18.659	4,5-di-epi-aristolochene	0.2	1474	Sesquiterpene
September	A	13	18.747	Aciphyllene	0.72	1490	Sesquiterpene

September	A	14	18.847	α-Panasinsene	0.21	1416	Sesquiterpene
September	A	15	19.166	Eudesma-4(14),11-diene	18.72	1469	Sesquiterpene
September	A	16	19.275	Longifolene	0.61	1398	Sesquiterpene
September	A	17	19.845	Guaia-1(10),11-diene	0.26	1490	Sesquiterpene
September	A	18	21.325	Caryophyllene oxide	0.29	1507	Oxygenated sesquiterpene
September	A	19	22.158	α-Cedrene epoxide	1.16	1293	Oxygenated sesquiterpene
September	A	20	32.331	Phytol	0.15	2045	Oxygenated diterpene
December	A	1	4.672	α-Pinene	0.17	948	Monoterpene
December	A	2	5.664	β-Pinene	0.17	943	Monoterpene
December	A	3	15.893	4,4-Dimethyl-3-(3-methylbut-3-enylidene)-2-methylenebicyclo[4.1.0]heptane	0.21	1392	Sesquiterpene
December	A	4	16.199	Dehydro Aromadendrene	0.25	1396	Sesquiterpene
December	A	5	16.431	Germacrene B	0.22	1570	Sesquiterpene
December	A	6	16.626	β-Elemene	3.43	1398	Sesquiterpene
December	A	7	16.907	τ-Elemene	1.01	1398	Sesquiterpene
December	A	8	17.184	α-Maaliene	0.34	1432	Sesquiterpene
December	A	9	17.400	cis-Caryophyllene	36.76	1494	Sesquiterpene
December	A	10	17.497	Thymohydroquinone dimethyl ether	26.4	1423	Oxygenated phenylpropanoid
December	A	11	18.120	Longicyclene	0.1	11.84	Sesquiterpene
December	A	12	18.293	α-Caryophyllene	0.58	1579	Sesquiterpene
December	A	13	18.658	4,5-di-epi-aristolochene	0.25	1474	Sesquiterpene
December	A	14	18.745	Aciphyllene	1.06	1490	Sesquiterpene
December	A	15	18.846	α-Panasinsene	0.3	1416	Sesquiterpene
December	A	16	19.188	Eudesma-4(14),11-diene	24.98	1469	Sesquiterpene

December	A	17	19.291	Guaia-1(10),11-diene	1.14	1490	Sesquiterpene
December	A	18	19.485	δ-Guaiene	0.25	1490	Sesquiterpene
December	A	19	19.849	Valencene	0.43	1474	Sesquiterpene
December	A	20	21.335	Caryophyllene oxide	0.27	1507	Oxygenated sesquiterpene
December	A	21	22.166	α-Cedrene epoxide	1.01	1293	Oxygenated sesquiterpene
December	A	22	23.129	Thunbergol	0.26	2211	Oxygenated diterpene
December	A	23	32.285	Phytol	0.41	2045	Oxygenated diterpene
